# Swine NONO promotes IRF3-mediated antiviral immune response by Detecting PRRSV N protein

**DOI:** 10.1371/journal.ppat.1012622

**Published:** 2024-10-16

**Authors:** Dandan Jiang, Chao Sui, Xiangju Wu, Ping Jiang, Juan Bai, Yue Hu, Xiaoyan Cong, Juntong Li, Dongwan Yoo, Laura C. Miller, Changhee Lee, Yijun Du, Jing Qi

**Affiliations:** 1 Shandong Key Laboratory of Animal Disease Control and Breeding/Key Laboratory of Livestock and Poultry Multi-omics of MARA, Institute of Animal Science and Veterinary Medicine, Shandong Academy of Agricultural Sciences, Jinan, Shandong, China; 2 Laboratory Animal Center, Qilu Hospital of Shandong University, Jinan, Shandong, China; 3 Key Laboratory of Animal Diseases Diagnostic and Immunology, Ministry of Agriculture, MOE International Joint Collaborative Research Laboratory for Animal Health & Food Safety, College of Veterinary Medicine, Nanjing Agricultural University, Nanjing, China; 4 Department of Pathobiology, College of Veterinary Medicine, University of Illinois at Urbana-Champaign, Urbana, Illinois, United States of America; 5 Department of Diagnostic Medicine and Pathobiology, College of Veterinary Medicine, Kansas State University, Manhattan, Kansas, United States of America; 6 College of Veterinary Medicine and Virus Vaccine Research Center, Gyeongsang National University, Jinju, Republic of Korea; University of Colorado Denver, UNITED STATES OF AMERICA

## Abstract

Non-POU domain-containing octamer-binding protein (NONO) is a multi-functional nuclear protein which belongs to the Drosophila behavior/human splicing (DBHS) protein family. NONO is known to regulate multiple important biological processes including host antiviral immune response. However, whether NONO can inhibit porcine reproductive and respiratory syndrome virus (PRRSV) replication is less well understood. In this study, we demonstrated that swine NONO (sNONO) inhibited PRRSV replication, via increasing expression of IFN-β, whereas NONO knockdown or knockout in PAM-KNU cells was more susceptible to PRRSV infection. As an IRF3 positive regulation factor, NONO promoted IFN-β expression by enhancing activation of IRF3. During PRRSV infection, NONO further up-regulated IRF3-mediated IFN-β expression by interacting with PRRSV N protein. Mechanistically, NONO functioned as a scaffold protein to detect PRRSV N protein and formed N-NONO-IRF3 complex in the nucleus. Interestingly, it was found that the NONO protein reversed the inhibitory effect of PRRSV N protein on type I IFN signaling pathway. Taken together, our study provides a novel mechanism for NONO to increase the IRF3-mediated IFN-β activation by interacting with the viral N protein to inhibit PRRSV infection.

## Author summary

Porcine reproductive and respiratory syndrome (PRRS), which is caused by porcine reproductive and respiratory syndrome virus (PRRSV), is a highly infectious disease and leads to huge economic losses to the global swine industry. Here, we reported that Non-POU domain-containing octamer-binding protein (NONO) significantly inhibited PRRSV replication and increased the expression of IFN-β. We found that NONO positively regulated IFN-β signaling pathway by targeting IRF3 and interacted with PRRSV N protein to promote activation of IFN-β, which reversed the inhibitory effect of PRRSV N protein on type I IFN signaling. We also found that NONO acted as a bridge to recruit PRRSV N and IRF3 to form N-NONO-IRF3 complex and enhanced activation of IFN-β signaling. Our study uncovers a novel mechanism for NONO to exert antiviral response through interacting with PRRSV N protein and IRF3 to inhibit PRRSV replication.

## Introduction

Porcine reproductive and respiratory syndrome virus (PRRSV) is the etiological agent of porcine reproductive and respiratory syndrome (PRRS) which causes substantial economic losses and has remained a top challenge to the pig industry. PRRSV belongs to the genus *Betaarterivirus* of the family *Arteriviridae* in the order *Nidovirales*. The genome of PRRSV is a single-stranded, positive-sense RNA of approximately 15kb in length which contains 10 open reading frames (ORFs) encoding two nonstructural polyproteins (pp1a and pp1ab) and eight structural proteins (GP2, E, GP3, GP4, GP5, ORF5a, M, and N). The pp1a and pp1ab are cleaved into at least 16 nonstructural proteins (nsps): nsp1α, nsp1β, nsp2, nsp2TF/nsp2N, nsp3 to nsp6, nsp7α, nsp7β, and nsp8 to nsp12 by virus-encoded proteases (nsp1α, nsp1β, nsp2, and nsp4). Previous studies have demonstrated several host proteins can inhibit PRRSV infection by regulating viral processes in multiple mechanisms, such as viral entry, viral protein synthesis, viral genome replication, and viral body release [1–3]. For example, the interferon-induced transmembrane protein 3 (IFITM3) induces accumulation of cholesterol in cellular vesicles to restrict PRRSV membrane fusion and prevent viral infection [4]. During PRRSV infection, cyclic GMP-AMP synthase (cGAS) senses mitochondrial DNA (mtDNA) and increases the production of cGAMP to restrict viral replication [5]. Cholesterol 25-hydroxylase (CH25H) decreases viral penetration and degrades nsp1α in the ubiquitin-proteasome pathway to antagonize PRRSV infection [6]. RING finger protein 114 (RNF114) promotes K27-linked polyubiquitination and degradation of nsp12 in a proteasome-dependent manner to suppress PRRSV replication [7].

Non-POU domain-containing octamer-binding protein (NONO), also known as 54-kDa nuclear RNA- and DNA- binding protein (p54^nrb^), is a multifunctional nuclear protein which belongs to the Drosophila behavior/human splicing (DBHS) protein family [8,9]. NONO is involved in many important biological processes, such as DNA repair, mRNA splicing, nuclear retention of defective RNA, and transcriptional regulation [10–12]. In addition, NONO plays a critical role in host antiviral innate immunity. Previous studies have suggested that NONO promotes cGAS-mediated antiviral response by interacting with the capsid protein to inhibit HIV infection [13]. In addition, the HIV-1 replication is restricted by NONO in CD4(+) T cells [14]. However, whether PRRSV infection is regulated by swine NONO remains unknown.

In the present study, we provide evidence that NONO promotes activation of IFN-β signaling pathway as a positive regulatory factor of IRF3. NONO promotes the formation of N-NONO-IRF3 complex and phosphorylation of IRF3 by association with PRRSV N protein in the nucleus, which further enhances the antiviral response of IFN-β. The NONO protein reverses the activity of PRRSV N in antagonizing the type I IFN signaling pathway. These data present a novel mechanism for NONO to positively regulate host antiviral response through interaction with PRRSV N protein and IRF3.

## Results

### Inhibition of PRRSV replication by swine NONO

**Fig 1:**
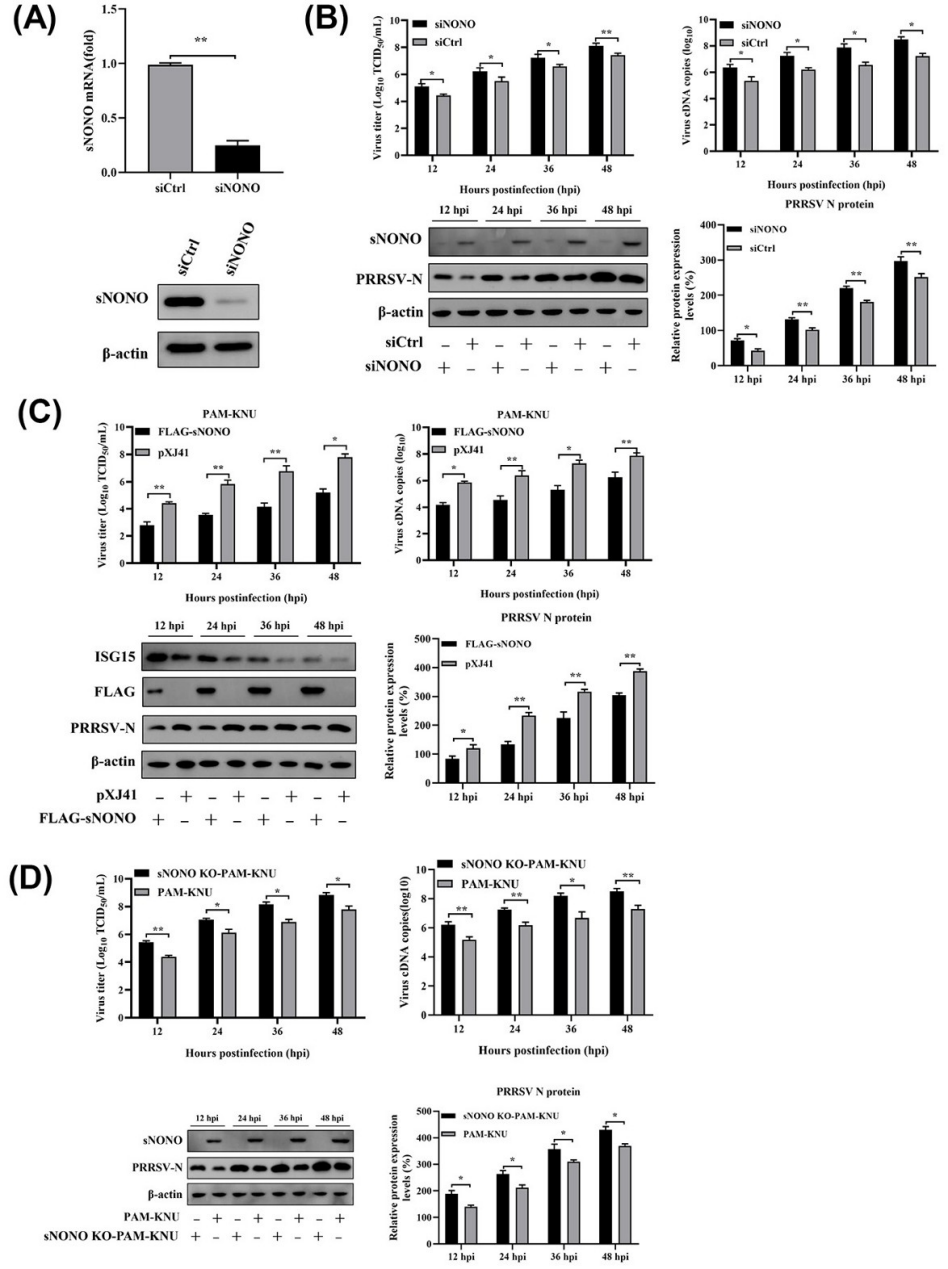
NONO inhibits PRRSV replication. (A) PAM-KNU cells were transfected with 10 pmol of control siRNA (siCtrl) or siRNA targeting NONO (siNONO) for 24 h. The mRNA levels of NONO were detected by real-time PCR (n = 3 independent experiment, **p < 0.01, bar indicates mean). The protein levels of NONO were detected by Western blotting with anti-NONO antibody. The same blot was incubated with β-actin antibody as a protein loading control (n = 3 independent experiment, one representative experiment is shown). (B-D) PAM-KNU cells were transfected with 10 pmol of siNONO or siCtrl for 24 h and then infected with HP-PRRSV SY0608 strain at an MOI of 1 (B). PAM-KNU cells were transfected with 1 μg of pXJ41-sNONO or pXJ41 for 24 h and infected with HP-PRRSV SY0608 strain at an MOI of 1 (C). PAM-KNU and sNONO KO-PAM-KNU cells were infected with HP-PRRSV SY0608 strain at an MOI of 1 (D). Culture supernatants were collected at indicated times and viral RNA was extracted from culture supernatants to analyze by real-time PCR. Virus titers in culture supernatants were measured by microtitration infectivity assay and calculated TCID_50_ using the Reed-Muench method (n = 3 independent experiment, *p < 0.05, **p < 0.01, bar indicates mean). Whole-cell lysates were immunoblotted with anti-FLAG, anti-NONO, anti-N, or anti-ISG15 antibody. The same blot was incubated with β-actin antibody as a protein loading control (n = 3 independent experiment, one representative experiment is shown). The band intensities of N are shown as the relative protein expression levels, normalized with β-actin (n = 3 independent experiment, *p < 0.05, **p < 0.01, bar indicates mean).

Previously we have reported that expression of NONO is significantly up-regulated in PRRSV-infected monocyte-derived dendritic cells (MoDCs) by iTRAQ-coupled 2D LC—MS/MS analysis [15]. To explore the possible role of NONO in the antiviral signaling pathway, we designed a small interfering RNA (siRNA) and transfected it into PAM-KNU cells. It was found that the NONO mRNA and protein levels were much lower in NONO specific siRNA (siNONO) transfected cells than those in control siRNA (siCtrl) transfected cells (Fig 1A). siRNA-mediated knockdown of NONO expression increased PRRSV titers, virus cDNA copies and N protein expression (Fig 1B). In contrast, overexpression of NONO in PAM-KNU and MARC-145 cells significantly decreased PRRSV titers, virus cDNA copies and N protein expression (Figs 1C and S1). Previous studies have reported that PRRSV infection significantly reduced ISG15 expression [16,17]. However, compared to pXJ41 transfected cells, the protein level of ISG15 was still increased in pXJ41-sNONO transfected PAM-KNU cells at an indicated time (Fig 1C). To further study the antiviral function of NONO, sNONO KO-PAM-KNU cell line with NONO knockout in PAM-KNU cells was generated by CRISPR-Cas9 gene editing system. In PRRSV-infected sNONO KO-PAM-KNU cells, the viral titers and cDNA copies were higher than those in PAM-KNU cells. Meanwhile, the PRRSV N protein level was significantly enhanced in sNONO KO-PAM-KNU cells (Fig 1D). In addition, PRRSV infection increased the protein level of NONO (Fig 1B and 1D). These results suggest that NONO can inhibit PRRSV replication.

### Positive regulation of IFN-β signaling pathway by NONO

**Fig 2:**
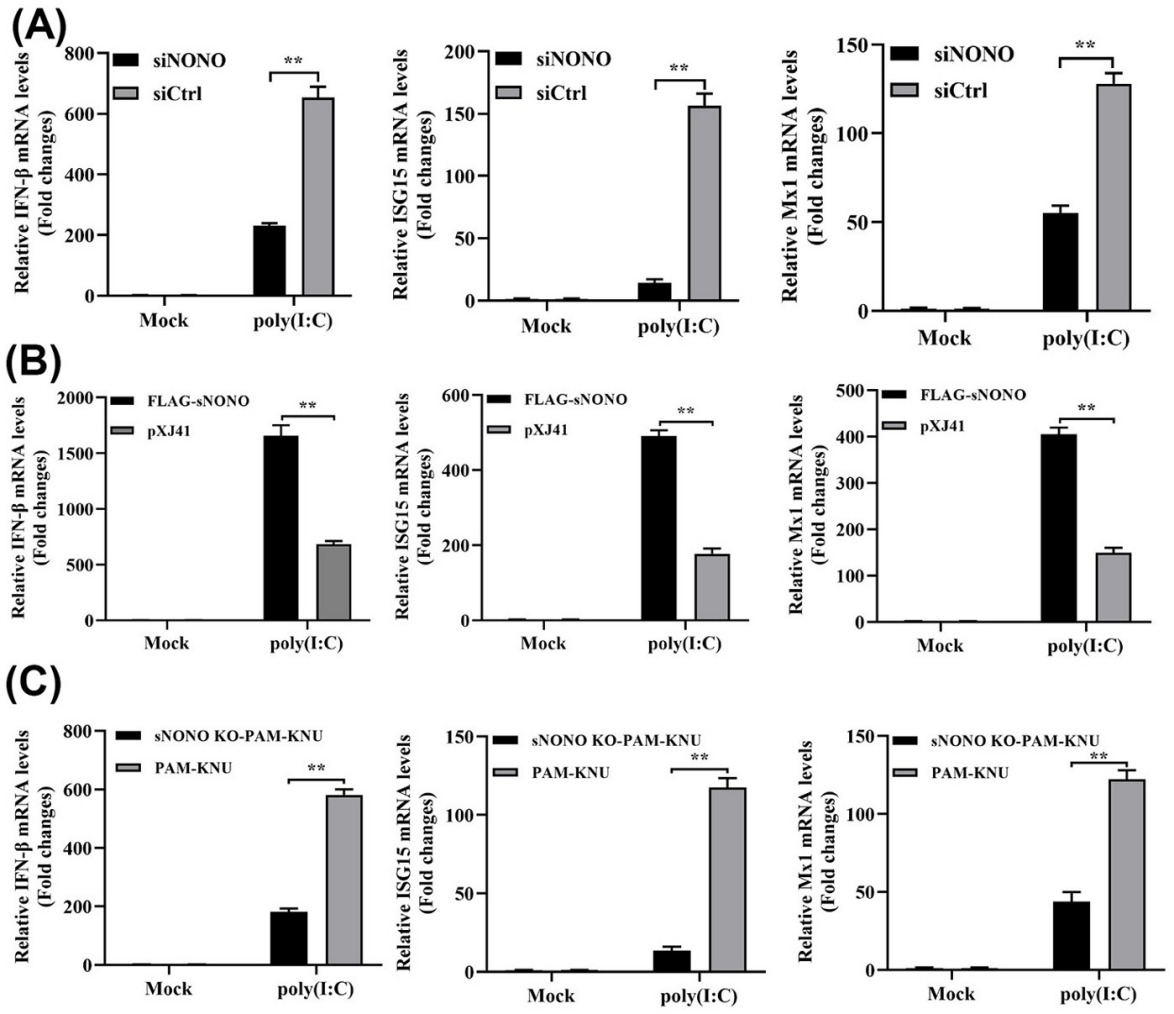
NONO positively regulates IFN-β signaling pathway. (A-C) PAM-KNU cells were transfected with 10 pmol of siNONO or siCtrl for 24 h and then treated with 1 μg/mL poly (I:C) for 6 h (A). PAM-KNU cells were transfected with 1 μg of pXJ41-sNONO or pXJ41 for 24 h and treated with 1 μg/mL poly (I:C) for 6 h (B). PAM-KNU and sNONO KO-PAM-KNU cells were treated with 1 μg/mL poly (I:C) for 6 h (C). Cells were harvested and total RNA was extracted using TRIzol reagent. The mRNA levels of IFN-β, ISG15 and Mx1 were analyzed by real-time PCR (n = 3 independent experiment, **p < 0.01, bar indicates mean).

Previous studies reported that NONO positively regulates cGAS-mediated IFN-β expression to inhibit HIV infection [13]. We hypothesize that NONO antagonizes PRRSV replication by influencing expression of IFN-β. Knockdown of NONO by siRNA transfected in PAM-KNU cells down-regulated mRNA levels of IFN-β, ISG15 and Mx1 induced by poly (I:C) (Fig 2A). Meanwhile, the IFN-β promoter activity induced by poly (I:C) or VSV was significantly lower in siNONO transfected PAM-KNU cells than those in siCtrl transfected PAM-KNU cells (S2A Fig). In contrast, real-time PCR analysis showed that mRNA levels of IFN-β, ISG15 and Mx1 induced by poly (I:C) in pXJ41-sNONO transfected cells were significantly higher than those in pXJ41 transfected PAM-KNU cells (Fig 2B). The IFN-β promoter activity was also enhanced in sNONO overexpressing cells induced by poly (I:C) or VSV (S2B Fig). In sNONO KO-PAM-KNU cells, the mRNA levels of IFN-β, ISG15, and Mx1 were markedly decreased in comparison to those in PAM-KNU cells (Fig 2C). IFN-β promoter activity stimulated by poly (I:C) or VSV was also decreased in sNONO KO-PAM-KNU cells (S2C Fig). These results reveal that NONO positively regulates activation of IFN-β signaling pathway induced by poly (I:C) and VSV.

### NONO positively regulates IFN-β signaling pathway by targeting IRF3

**Fig 3:**
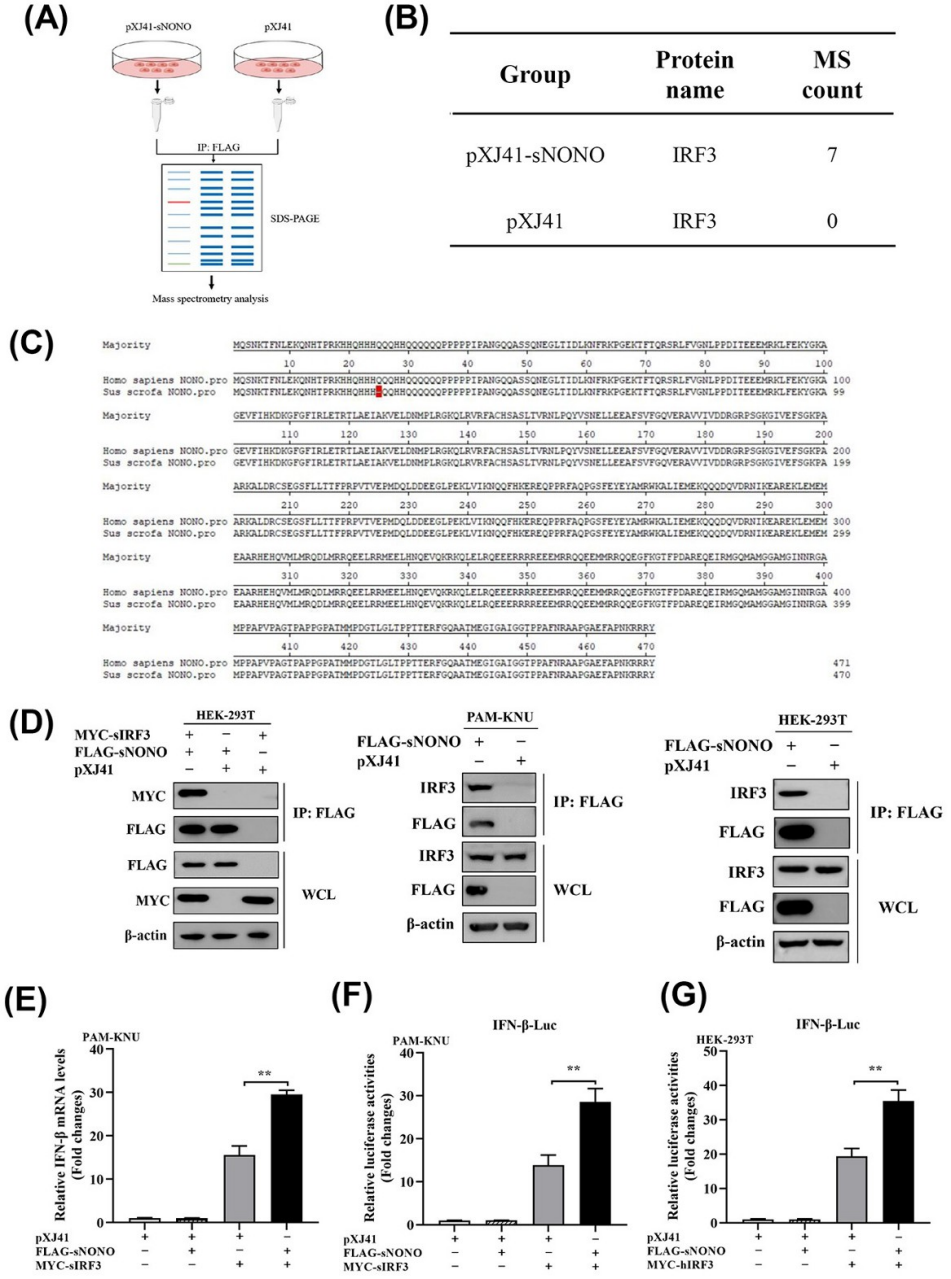
NONO interacts with IRF3 and promotes IRF3-mediated IFN-β signaling pathway. (A) PAM-KNU cells were transfected with 6 μg of pXJ41-sNONO or pXJ41 for 24 h. Then NONO complex was pulled down by immunoprecipitation with anti-FLAG antibody. The precipitation was subjected to SDS-PAGE and stained with Coomassie Blue, followed by analysis using protein affinity purification mass spectrometry (AP-MS). (B) Significant IRF3 enriched in NONO immunoprecipitates by quantitative AP-MS (n = 3 independent experiments). (C) The sequence alignment between swine and human NONO. (D) HEK-293T or PAM-KNU cells were cotransfected with 3 μg of pXJ41-sNONO and 3 μg of pXJ41-sIRF3 or pXJ41 for 24 h, followed by co-IP with anti-FLAG antibody and immunoblot analysis with anti-FLAG, anti-MYC or anti-IRF3 antibody. Whole-cell lysis (WCL) was subjected to Western blotting using anti-FLAG, anti-MYC, anti-IRF3, or anti-β-actin antibody (n = 3 independent experiment, one representative experiment is shown). (E) PAM-KNU cells were transfected with 0.5 μg of pXJ41-sNONO and 0.5 μg of pXJ41-sIRF3 or pXJ41 for 24 h. The cells were harvested and total RNA was extracted by TRIzol reagent. The mRNA level of IFN-β was analyzed by real-time PCR (n = 3 independent experiment, **p < 0.01, bar indicates mean). (F) PAM-KNU cells were transfected with 0.3 μg of pXJ41-sNONO and 0.3 μg of pXJ41-sIRF3 or pXJ41, along with 0.3 μg of pIFN-β-Luc and 0.03 μg of pRL-TK luciferase reporter for 24 h. IFN-β promoter activity was detected by a dual-luciferase reporter assay (n = 3 independent experiment, **p < 0.01, bar indicates mean). (G) HEK-293T cells were transfected with 0.3 μg of pXJ41-sNONO and 0.3 μg of pXJ41-hIRF3 or pXJ41, along with 0.3 μg of pIFN-β-Luc and 0.03 μg of pRL-TK luciferase reporter for 24 h. IFN-β promoter activity was detected by a dual-luciferase reporter assay (n = 3 independent experiment, **p < 0.01, bar indicates mean).

To identify the adaptor molecule regulated by NONO in the IFN-β signaling pathway, we used affinity purification mass spectrometry (AP-MS) to analyze the NONO binding proteins. PAM-KNU cells were transfected with pXJ41-sNONO or pXJ41 for 24 h. The NONO complex was pulled down by immunoprecipitation with anti-FLAG antibody, and the samples were subjected to SDS-PAGE and stained with coomassie blue, followed by mass spectrometry analysis (Fig 3A). It was found that IRF3 peptides were enriched in the pXJ41-sNONO transfected group rather than in pXJ41 transfected group (Fig 3B and S1 Table), indicating that IRF3 probably plays a critical role in the NONO-mediated IFN-β signaling pathway as an adaptor candidate. NONO, as a nuclear protein, is able to regulate activation of many transcriptional factors [18,19]. IRF3 is a transcription factor of the IFN-β signaling pathway, and we have hypothesized that NONO promoted IFN-β expression by regulating activation of IRF3. The homology analysis showed that there was only one amino acid difference between swine and human NONO sequences (Fig 3C). Co-immunoprecipitation (Co-IP) experiments showed that NONO interacted with IRF3 in both human and swine cells (Figs 3D and S3A). NONO knockout did not affect protein levels of IRF3 and p65 (S3B Fig). The mRNA level of IFN-β in pXJ41-sNONO transfected PAM-KNU cells was significantly increased in comparison with pXJ41 transfected cells after IRF3 induction (Fig 3E). Meanwhile, in PAM-KNU and HEK-293T cells, overexpression of NONO enhanced IFN-β promoter activity induced by swine or human IRF3 (Fig 3F and 3G). These results suggest that NONO positively regulates the IFN-β signaling pathway by targeting IRF3.

### NONO interacts with PRRSV N protein

**Fig 4:**
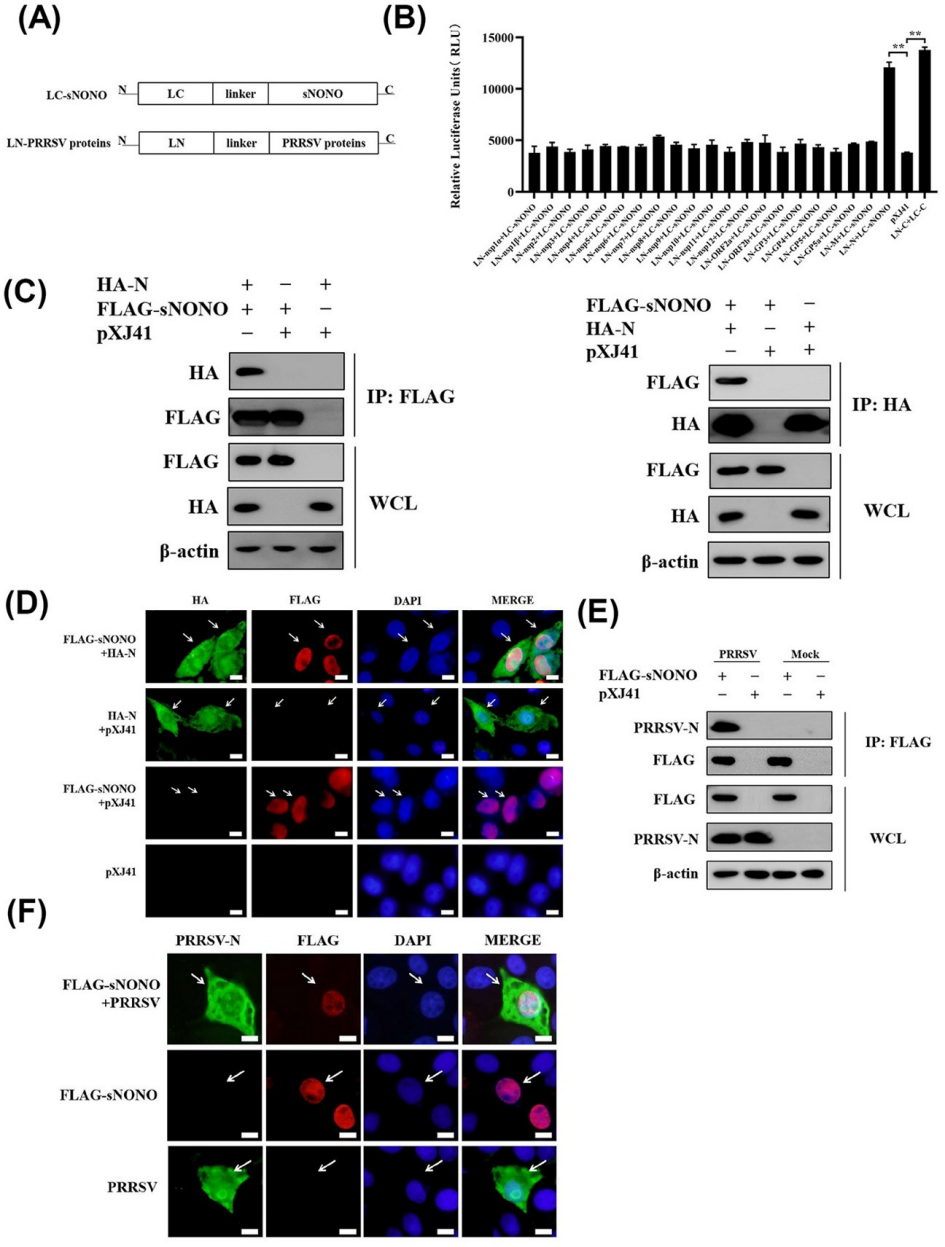
NONO interacts with PRRSV N protein. (A) Schematic representation of LC-NONO and LN-PRRSV proteins. (B) HEK-293T cells were cotransfected with 0.5 μg of LC-NONO and 0.5 μg of LN-PRRSV constructs for 24 h. Cells were harvested and analyzed for recombination *Renilla* luciferase reporter activity by luciferase assay system (n = 3 independent experiment, **p < 0.01, bar indicates mean). (C) HEK-293T cells were cotransfected with 3 μg of pXJ41-sNONO and 3 μg of pXJ41-N or pXJ41 for 24 h. Cells were harvested and subjected to co-IP with anti-FLAG or anti-HA antibody. Immunocomplexes were analyzed by Western blotting using anti-HA or anti-FLAG antibody. WCL was also subjected to Western blotting using anti-HA, anti-FLAG, or anti-β-actin antibody (n = 3 independent experiment, one representative experiment is shown). (D) PAM-KNU cells were cotransfected with 0.25 μg of pXJ41-sNONO and 0.25 μg of pXJ41-N or pXJ41 for 18 h. Indirect immunofluorescence (IFA) was performed using anti-HA antibody (green), anti-FLAG antibody (red), or DAPI (blue). Scale bar, 10 μm. (E) PAM-KNU cells were transfected with 6 μg of pXJ41-sNONO or pXJ41 for 18 h and then infected with PRRSV at an MOI of 1 for 12 h. Cells were harvested and subjected to co-IP with anti-FLAG antibody. Immunocomplexes were analyzed by Western blotting using anti-N antibody. WCL was also subjected to Western blotting using anti-FLAG, anti-N, or anti-β-actin antibody (n = 3 independent experiment, one representative experiment is shown). (F) PAM-KNU cells were transfected with 0.25 μg of pXJ41-sNONO or pXJ41 for 18 h and infected with PRRSV at an MOI of 1 for 12 h. Indirect immunofluorescence (IFA) was performed using anti-N antibody (green), anti-FLAG antibody (red), or DAPI (blue). Scale bar, 10 μm.

To identify a viral protein regulated by NONO during PRRSV infection, we generated a series of SLCA (split luciferase complementation assay) constructs for PRRSV proteins and sNONO (Fig 4A). The relative luciferase units (RLU) were detected in HEK-293T cells transfected with LC-sNONO plasmid and individual plasmid expressing LN-PRRSV proteins. The pair of LC-C and LN-C containing Zika virus (ZIKV) C protein was used as a positive control and pXJ41 was used as a negative control. The RLU of LC-sNONO and LN-N transfected group was significantly higher than those of other groups except for positive control (Fig 4B), indicating the PRRSV N protein is a binding partner for sNONO.

To further determine the interaction of NONO and PRRSV N, HEK-293T cells were cotransfected with pXJ41-sNONO and pXJ41-N. Co-IP experiments showed that sNONO interacted with PRRSV N (Fig 4C). FLAG-sNONO colocalized with PRRSV HA-N in the nucleus concurrently (Fig 4D). To further confirm that NONO associates with PRRSV N protein, PAM-KNU cells were transfected with pXJ41-sNONO and then infected with PRRSV for 12 h. IP with anti-NONO antibody followed by immunoblotting with anti-N antibody showed that FLAG-sNONO interacted with N protein in PRRSV infected cells (Fig 4E). Furthermore, there was colocalization of NONO and N protein in the nucleus after PRRSV infection (Fig 4F). Meanwhile, NONO knockout did not affect PRRSV N protein localization in pXJ41-N transfected or PRRSV infected cells (S4 Fig). These results demonstrate that NONO protein interacts and colocalizes with PRRSV N protein.

**Fig 5:**
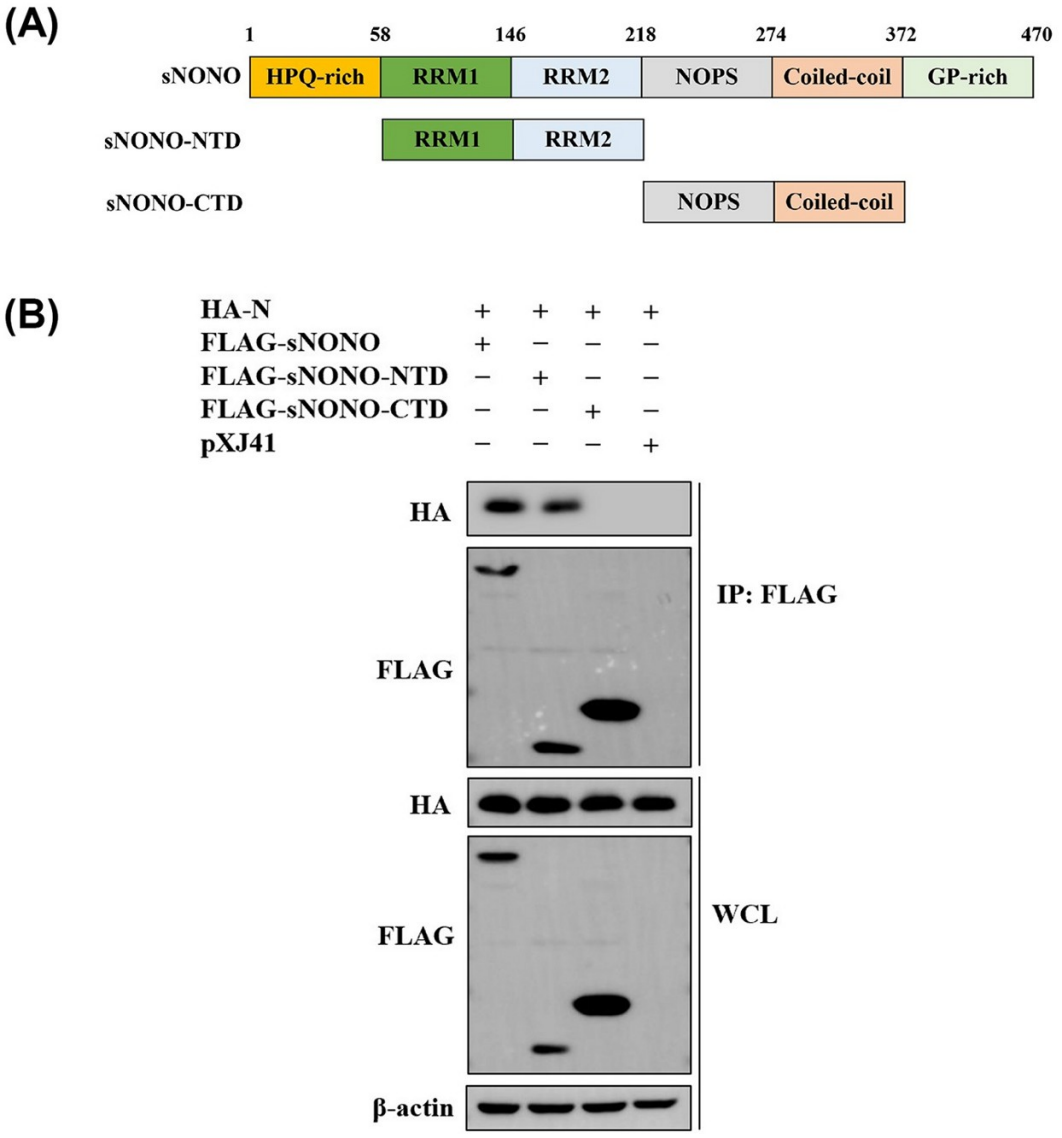
PRRSV N associates with N-terminal domain of NONO. (A) Schematic representation of swine NONO structure. (B) HEK-293T cells were cotransfected with 3 μg of pXJ41-N and 3 μg of pXJ41-sNONO, pXJ41-sNONO-NTD or pXJ41-sNONO-CTD for 24 h. Cells were harvested and subjected to co-IP with anti-FLAG antibody. Immunocomplexes were analyzed by Western blotting using anti-HA antibody. WCL was also subjected to Western blotting using anti-HA, anti-FLAG, or anti-β-actin antibody (n = 3 independent experiment, one representative experiment is shown).

**Fig 6:**
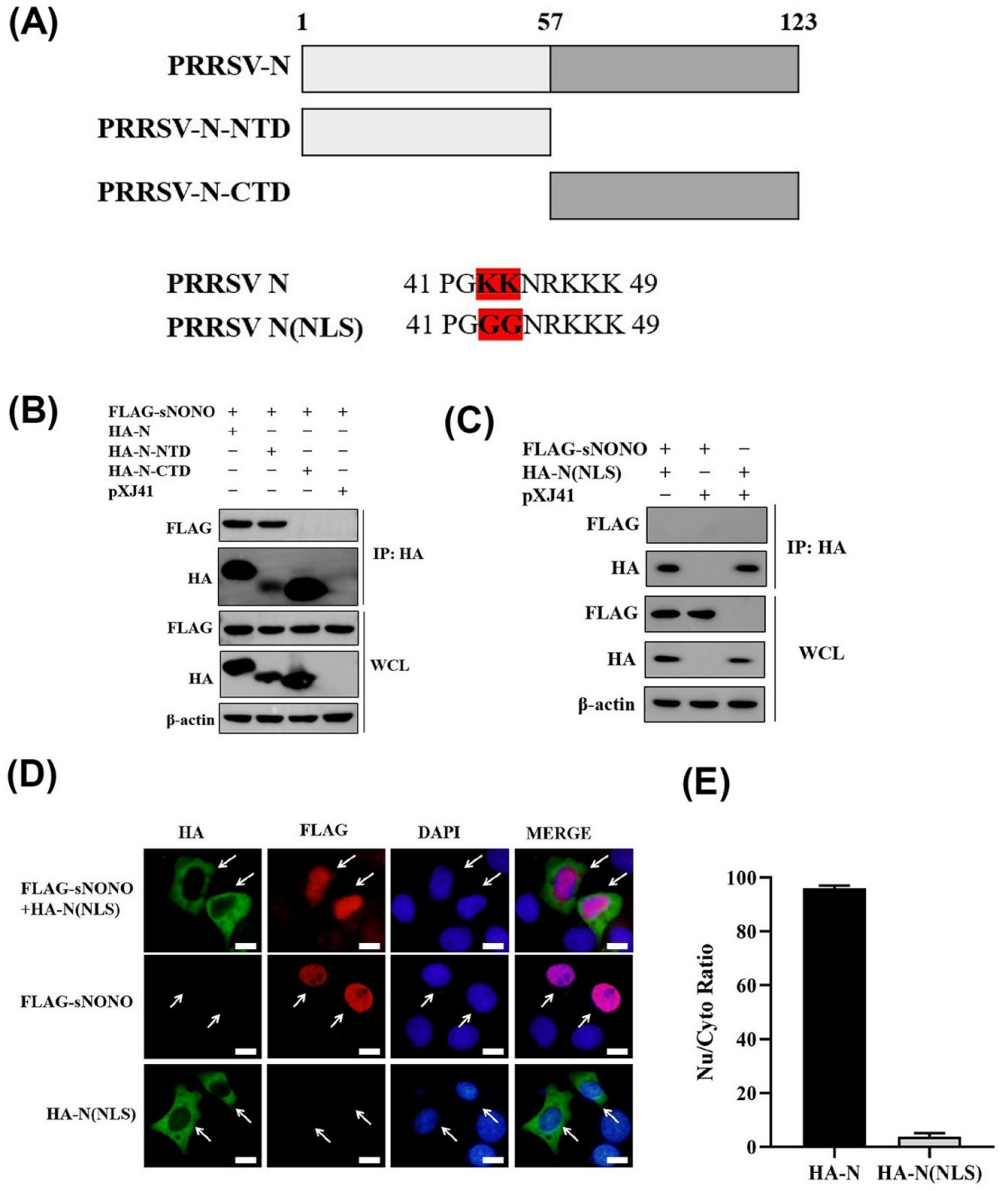
NONO interacts with N-terminal domain of PRRSV N. (A) Schematic representation of PRRSV N structure. (B) HEK-293T cells were cotransfected with 3 μg of pXJ41-sNONO and 3 μg of pXJ41-N, pXJ41-N-NTD or pXJ41-N-CTD for 24 h. Cells were harvested and subjected to co-IP with anti-HA antibody. Immunocomplexes were analyzed by Western blotting using anti-FLAG antibody. WCL was also subjected to Western blotting using anti-HA, anti-FLAG, or anti-β-actin antibody (n = 3 independent experiment, one representative experiment is shown). (C) HEK-293T cells were cotransfected with 3 μg of pXJ41-sNONO and 3 μg of pXJ41-N(NLS) or pXJ41 for 24 h. Cells were harvested and subjected to co-IP with anti-HA antibody. Immunocomplexes were analyzed by Western blotting using anti-FLAG antibody. WCL was also subjected to Western blotting using anti-HA, anti-FLAG, or anti-β-actin antibody (n = 3 independent experiment, one representative experiment is shown). (D) PAM-KNU cells were cotransfected with 0.25 μg of pXJ41-sNONO and 0.25 μg of pXJ41-N(NLS) or pXJ41 for 18 h. Indirect immunofluorescence (IFA) was performed using anti-HA antibody (green), anti-FLAG antibody (red), or DAPI (blue). Scale bar, 10 μm. (E) Quantitative measurement of the nuclear/cytosolic ratio of N in Fig 4D or N(NLS) in Fig 6D. The number of N or N(NLS) nuclear localization cells was determined by counting 100 cells each in random microscopic fields (n = 3 independent experiments, bar indicates mean).

Based on the structure of NONO and PRRSV N protein, we generated four plasmids encoding the N-terminal domain (NTD) and C-terminal domain (CTD) of NONO and N protein, respectively (Figs 5A and 6A). The pXJ41-sNONO-NTD construct contained the N-terminal 58 to 217 amino acids of NONO and pXJ41-sNONO-CTD construct contained the C-terminal 218 to 371 amino acids of NONO (Fig 5A). Co-IP experiments showed that N-terminal domain of NONO interacted with PRRSV N protein (Fig 5B). The pXJ41-N-NTD construct contained the N-terminal 1 to 56 amino acids of N and pXJ41-N-CTD construct contained the C-terminal 57 to 123 amino acids of N. The pXJ41-N(NLS) plasmid encoded PRRSV N protein containing two specific mutations (K43G and K44G) in the nuclear localization signal (NLS) sequence (Fig 6A). The results of co-IP experiments demonstrated that NONO interacted with the N-terminal domain of N protein (Fig 6B). In addition, the NLS of N was a key sequence for interaction of NONO and N (Fig 6C). The colocalization between the mutant construct N(NLS) and NONO was not observed and NONO localization was not affected by N(NLS) (Fig 6D). N(NLS) was obtained by K43G and K44G specific mutations, which led to N protein localizing in the cytoplasm (Fig 6E). There was no interaction between N(NLS) and NONO, K43 and K44 were the key sites for interaction between NONO and PRRSV N protein. These data demonstrate that NONO interacts with N protein through N-terminal domain of NONO and N-terminal domain of N protein. Furthermore, the K43 and K44 in NLS of N protein is critical for their interaction.

### NONO interacts with PRRSV N protein to promote activation of IFN-β signaling pathway

**Fig 7:**
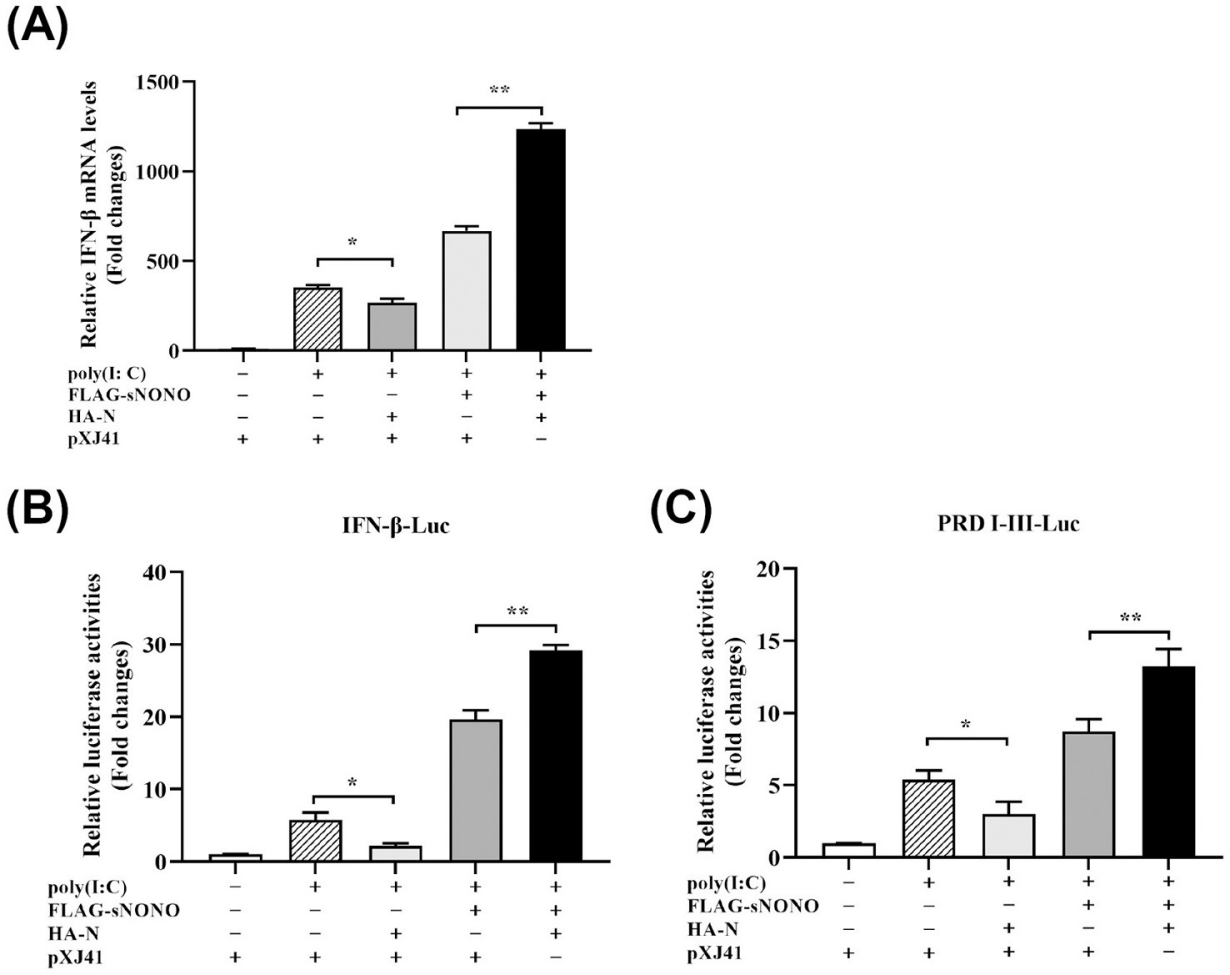
PRRSV N positively regulates IFN-β signaling pathway by interaction with NONO. (A) PAM-KNU cells were cotransfected with 1 μg of pXJ41-sNONO and 1 μg of pXJ41-N or pXJ41 for 24 h and treated with 1 μg/mL poly (I:C) for 6 h. Cells were harvested and total RNA was extracted. The mRNA level of IFN-β was analyzed using real-time PCR (n = 3 independent experiment, *p < 0.05, **p < 0.01, bar indicates mean). (B-C) HEK-293T cells were cotransfected with 0.3 μg of pXJ41-sNONO and 0.3 μg of pXJ41-N or pXJ41, along with 0.3 μg of pIFN-β-Luc or pPRDI-III-Luc and 0.03 μg of pRL-TK luciferase reporter for 24 h, and then treated with 1 μg/mL poly (I:C) for 6 h. Cells were harvested and IFN-β promoter (B) or PRD I-III promoter (C) activity was analyzed by a dual-luciferase reporter assay (n = 3 independent experiment, *p < 0.05, **p < 0.01, bar indicates mean).

In this study, we found that NONO positively regulates activation of IFN-β signaling pathway (Figs 2 and S2). However, it was reported that PRRSV N protein is a negative regulator for IFN-β production [20]. To study the effect of PRRSV N on NONO-mediated antiviral response, PAM-KNU cells were transfected with pXJ41-sNONO and pXJ41-N or pXJ41 and then treated with poly (I:C) to activate the IFN signaling. Real-time PCR analysis showed that the mRNA level of IFN-β was significantly increased in pXJ41-sNONO and pXJ41-N cotransfected cells compared to those in pXJ41-sNONO transfected cells (Fig 7A). In pXJ41-sNONO and pXJ41-N cotransfected HEK-293T cells, the IFN-β promoter and PRD I-III promoter activities were higher than those in pXJ41-sNONO transfected cells (Fig 7B and 7C). As compared to poly (I:C) positive control, PRRSV N protein could significantly down-regulate IFN-β production (Fig 7A, 7B and 7C), which was consistent with the previous report [20]. Interestingly, we found that the mRNA level of IFN-β, IFN-β promoter and PRD I-III promoter activities in pXJ41-sNONO and pXJ41-N cotransfected group were the highest, suggesting that NONO reversed the activity of PRRSV N protein in antagonizing type I interferon signaling pathway. These results show that NONO interacts with PRRSV N protein to promote the activation of IFN-β signaling pathway.

### NONO promotes IRF3-induced IFN-β expression through binding to PRRSV N protein

**Fig 8:**
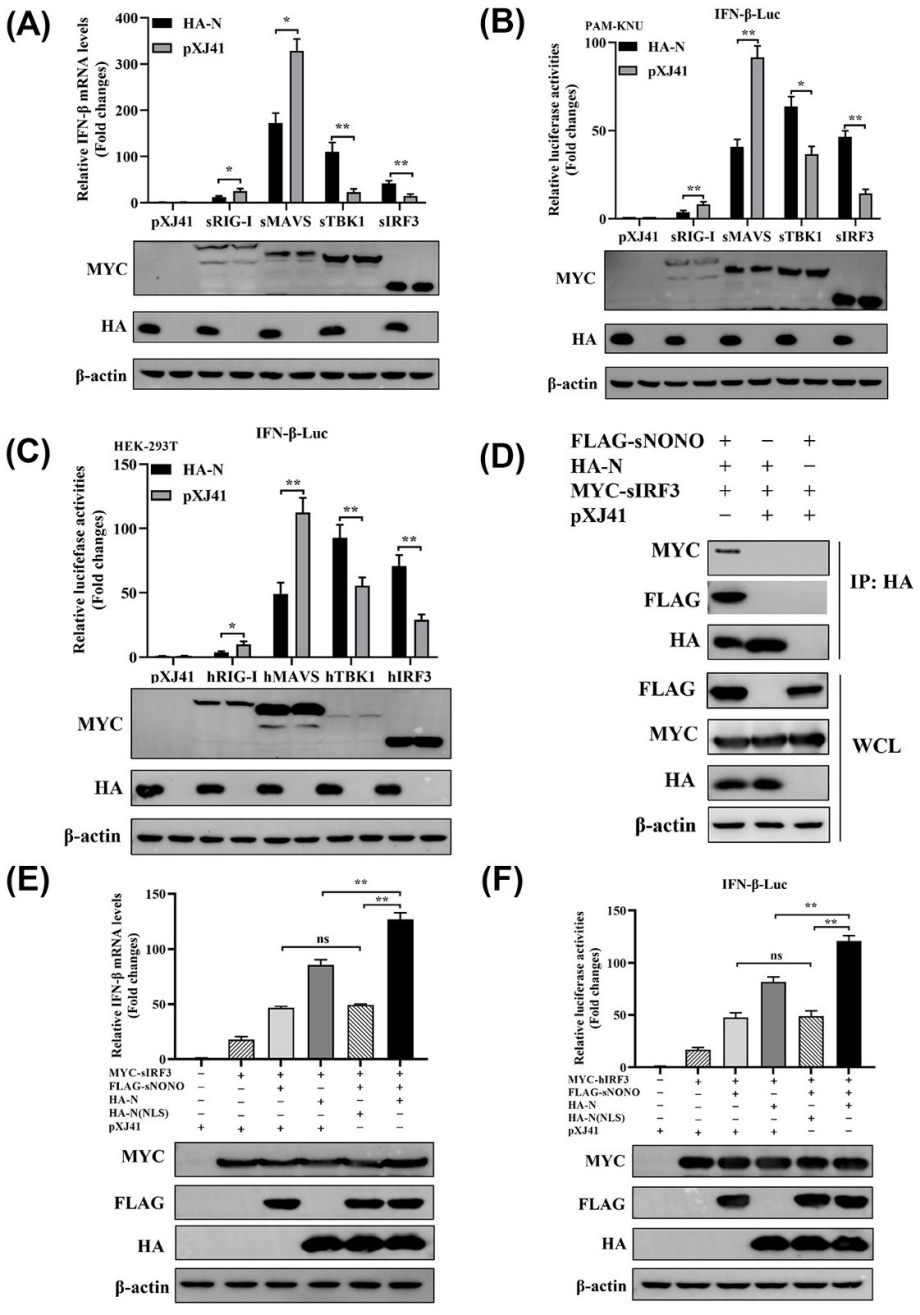
NONO promotes IRF3-induced IFN-β expression through binding to PRRSV N protein. (A) PAM-KNU cells were transfected with 0.5 μg of pXJ41-N or pXJ41 for 24 h, along with 0.5 μg of pXJ41-sRIG-I, pXJ41-sMAVS, pXJ41-sTBK1, or pXJ41-sIRF3. Cells were harvested and total RNA was extracted. The mRNA level of IFN-β was analyzed using real-time PCR (n = 3 independent experiment, *p < 0.05, **p < 0.01, bar indicates mean). Whole-cell lysates were immunoblotted with anti-MYC or anti-HA antibody. The same blot was incubated with β-actin antibody as a protein loading control (n = 3 independent experiment, one representative experiment is shown). (B) PAM-KNU cells were transfected with 0.3 μg of pXJ41-N or pXJ41 together with 0.3 μg of pXJ41-sRIG-I, pXJ41-sMAVS, pXJ41-sTBK1, or pXJ41-sIRF3 with 0.3 μg of pIFN-β-Luc and 0.03 μg of pRL-TK luciferase reporter for 24 h. The activation of IFN-β promoter was detected using a dual-luciferase reporter assay (n = 3 independent experiment, *p < 0.05, **p < 0.01, bar indicates mean). Whole-cell lysates were immunoblotted with anti-MYC or anti-HA antibody. The same blot was incubated with β-actin antibody as a protein loading control (n = 3 independent experiment, one representative experiment is shown). (C) HEK-293T cells were transfected with 0.3 μg of pXJ41-N or pXJ41 together with 0.3 μg of pXJ41-hRIG-I, pXJ41-hMAVS, pXJ41-hTBK1, or pXJ41-hIRF3 with 0.3 μg of pIFN-β-Luc and 0.03 μg of pRL-TK luciferase reporter for 24 h. The IFN-β promoter activity was detected using a dual-luciferase reporter assay (n = 3 independent experiment, *p < 0.05, **p < 0.01, bar indicates mean). Whole-cell lysates were immunoblotted with anti-MYC or anti-HA antibody. The same blot was incubated with β-actin antibody as a protein loading control (n = 3 independent experiment, one representative experiment is shown). (D) HEK-293T cells were transfected with 2 μg of pXJ41-N, 2 μg of pXJ41-sNONO and 2 μg of pXJ41-sIRF3 for 24 h. Cells were harvested and subjected to co-IP with anti-HA antibody. Immunocomplexes were analyzed by Western blotting using anti-HA, anti-FLAG and anti-MYC antibodies. WCL was also subjected to Western blotting using anti-HA, anti-FLAG, anti-MYC, or anti-β-actin antibody (n = 3 independent experiment, one representative experiment is shown). (E) PAM-KNU cells were cotransfected with 0.5 μg of pXJ41-sNONO and 0.5 μg of pXJ41-N, pXJ41-N(NLS) or pXJ41 for 24 h, along with 0.5 μg of pXJ41-sIRF3. Cells were harvested and total RNA was extracted. The mRNA level of IFN-β was analyzed using real-time PCR (n = 3 independent experiment, **p < 0.01, "ns" stands for not statistically significant, bar indicates mean). Whole-cell lysates were immunoblotted with anti-MYC, anti-FLAG, or anti-HA antibody. The same blot was incubated with β-actin antibody as a protein loading control (n = 3 independent experiment, one representative experiment is shown). (F) HEK-293T cells were cotransfected with 0.3 μg of pXJ41-sNONO and 0.3 μg of pXJ41-N, pXJ41-N(NLS) or pXJ41 together with 0.3 μg of pXJ41-sIRF3 for 24 h, along with 0.3 μg of pIFN-β-Luc and 0.03 μg of pRL-TK luciferase reporter. Cells were harvested and IFN-β promoter activity was analyzed by a dual-luciferase reporter assay (n = 3 independent experiment, **p < 0.01, "ns" stands for not statistically significant, bar indicates mean). Whole-cell lysates were immunoblotted with anti-MYC, anti-FLAG, or anti-HA antibody. The same blot was incubated with β-actin antibody as a protein loading control (n = 3 independent experiment, one representative experiment is shown).

To investigate the effect of NONO on IFN-β expression after PRRSV infection, PAM-KNU cells were transfected with pXJ41-N along with pXJ41-sRIG-I, pXJ41-sMAVS, pXJ41-sTBK1 or pXJ41-sIRF3. Real-time PCR analysis found that PRRSV N promoted IFN-β expression induced by sTBK1 and sIRF3 (Fig 8A). Meanwhile, IFN-β promoter activity was significantly enhanced in pXJ41-TBK1 or pXJ41-IRF3 transfected cells rather than in pXJ41-RIG-I or pXJ41-MAVS transfected PAM-KNU cells (Fig 8B) and HEK-293T cells (Fig 8C). Furthermore, N(NLS) inhibited RIG-I- and MAVS- mediated IFN-β expression but failed to increase TBK1- and IRF3-mediated IFN-β expression (S5A and S5B Fig). These data suggest that PRRSV N instead of N(NLS) promotes the TBK1- and IRF3-mediated IFN-β expression. Co-IP experiment results showed that PRRSV N protein did not directly bind to TBK1 or IRF3 in pXJ41-N and pXJ41-sTBK1 or pXJ41-sIRF3 transfected HEK-293T cells (S6A and S6B Fig). As a critical transcriptional factor, IRF3 is phosphorylated at its C-terminal regulatory domain, then dimerizes and translocates into nucleus to regulate production of type I interferon [21]. Thus, we detected the interaction between PRRSV N and IRF3 in pXJ41-N, pXJ41-sNONO and pXJ41-sIRF3 transfected HEK-293T cells (Fig 8D). The results showed that PRRSV N could interact with NONO, and NONO could interact with IRF3, suggesting that PRRSV N, NONO and IRF3 is able to form N-NONO-IRF3 complex. To further identify the function of PRRSV N protein in NONO-mediated IFN-β expression, pXJ41-sNONO and pXJ41-N were transfected into HEK-293T cells and IFN-β signaling pathway was induced by IRF3. The IFN-β mRNA level was significantly enhanced in pXJ41-sNONO and pXJ41-N cotransfected cells in comparison with pXJ41-sNONO transfected cells. However, N(NLS) failed to increase the IFN-β mRNA level (Fig 8E). Compared to pXJ41 transfected cells, the activities of IFN-β and PRD I-III promoters were increased in pXJ41-sNONO transfected cells. In pXJ41-sNONO and pXJ41-N cotransfected cells, the IFN-β promoter and PRD I-III promoter activities were much higher than those in NONO overexpressing cells. However, N(NLS) failed to enhance NONO-mediated IFN-β promoter or PRD I-III promoter activities (Figs 8F and S7). These data suggest that NONO further promotes IRF3-induced IFN-β expression through binding to PRRSV N protein.

### NONO enhances phosphorylation of IRF3 and interaction with IRF3 through association with PRRSV N

**Fig 9:**
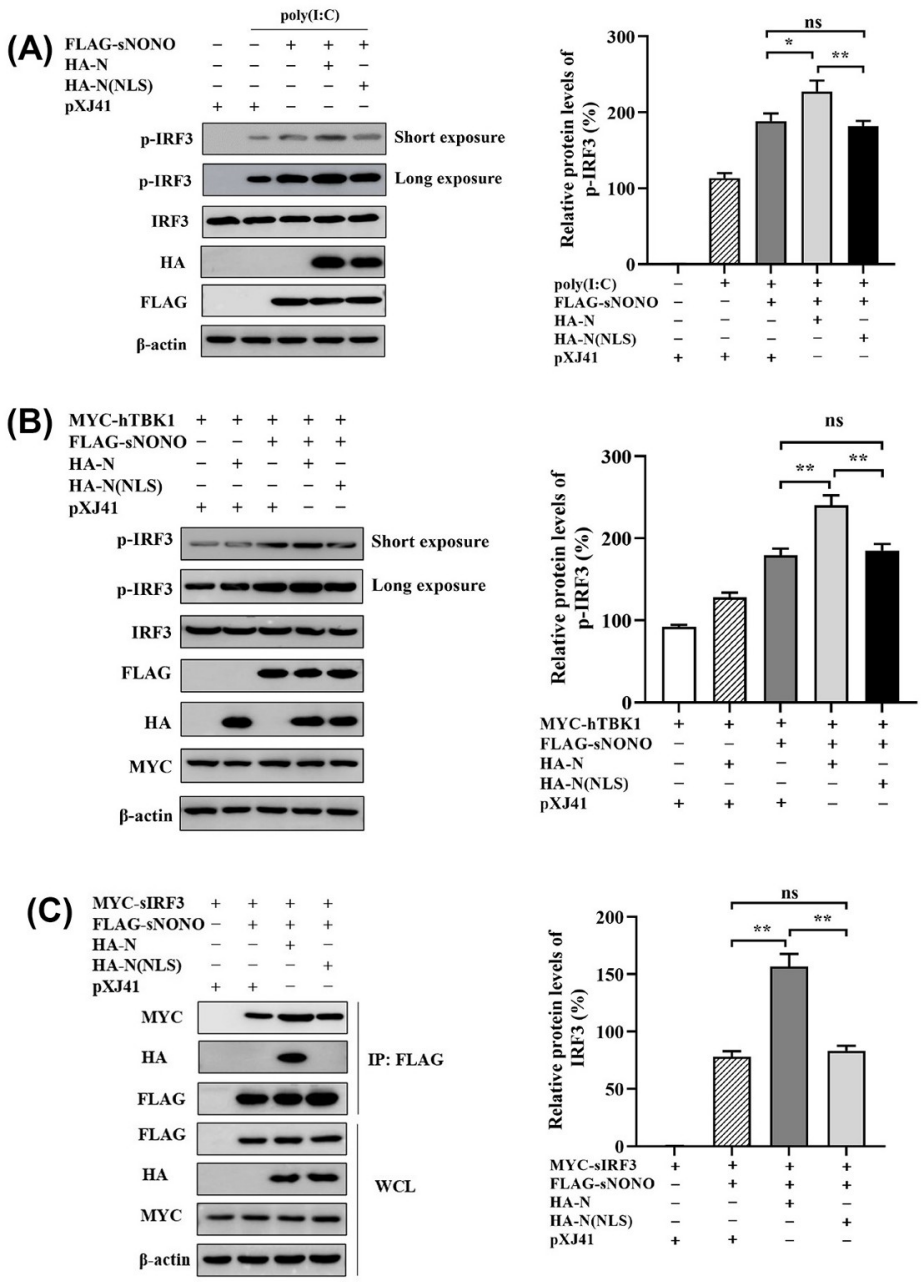
NONO enhances phosphorylation of IRF3 and interaction with IRF3 by binding to PRRSV N. (A-B) HEK-293T cells were cotransfected with 1 μg of pXJ41-sNONO and pXJ41-N, pXJ41-N(NLS), or pXJ41 for 24 h, along with 1 μg/mL poly (I:C) treated for 6 h (A) or 1 μg of pXJ41-hTBK1 transfected (B). Cell lysates were analyzed by Western blotting using antibody against p-IRF3, IRF3, HA, FLAG, or β-actin (n = 3 independent experiment, one representative experiment is shown). The band intensities of p-IRF3 (long exposure) are shown as the relative protein expression levels, normalized with β-actin (n = 3 independent experiment, *p < 0.05, **p < 0.01, "ns" stands for not statistically significant, bar indicates mean). (C) HEK-293T cells were cotransfected with 2 μg of pXJ41-sNONO and 2 μg of pXJ41-N, pXJ41-N(NLS), or pXJ41 for 24 h, along with 2 μg of pXJ41-sIRF3. Cells were harvested and subjected to co-IP with anti-FLAG antibody. Immunocomplexes were analyzed by Western blotting using anti-MYC, anti-FLAG or anti-HA antibody. WCL was also subjected to Western blotting using anti-FLAG, anti-HA, anti-MYC, or anti-β-actin antibody (n = 3 independent experiment, one representative experiment is shown). The band intensities of IRF3 are shown as the relative protein expression levels, normalized with β-actin (n = 3 independent experiment, **p < 0.01, "ns" stands for not statistically significant, bar indicates mean).

To further investigate the regulation mechanism of NONO protein on IFN-β signaling pathway, HEK-293T cells were cotransfected with pXJ41-sNONO and pXJ41-N or pXJ41-N(NLS). Compared to pXJ41-sNONO transfected cells, phosphorylation of IRF3 was much higher in pXJ41-sNONO and pXJ41-N cotransfected cells induced by poly (I:C) (Fig 9A). Meanwhile, in TBK1 transfected cells, coexpression of sNONO and PRRSV N protein significantly increased phosphorylation of IRF3 in comparison with sNONO alone expressing cells (Fig 9B). To further indicate the effect of N protein on the association of NONO and IRF3, HEK-293T cells were cotransfected with pXJ41-sNONO, pXJ41-sIRF3 and pXJ41-N. Co-IP experiments showed that N protein promoted the association of NONO and IRF3 (Fig 9C). These results further demonstrate that NONO acts as a bridge to recruit PRRSV N and IRF3 to form N-NONO-IRF3 complex and enhances activation of the IFN-β signaling pathway.

The NLS of PRRSV N protein plays a key role for viral RNA replication (31), and in our data, the N(NLS) failed to enhance phosphorylation of IRF3 induced by poly (I:C) and TBK1 in comparison with N protein (Fig 9A and 9B). At the same time, the interaction of NONO and IRF3 was not increased in N(NLS) expressing cells rather than in N protein expressing cells (Fig 9C), suggesting that NLS of PRRSV N protein is the key motif for NONO-mediated IFN-β expression. As N(NLS) was obtained by K43G and K44G specific mutations, K43 and K44 were the key sites for activation of IFN-β signaling pathway regulated by NONO. These results show that NONO binding to PRRSV N protein promotes activation of the IFN-β signaling pathway in the nucleus.

## Discussion

Type I interferons (IFN-α/β) constitute the innate immune defense of the host against viral infection [22]. Previous studies have reported that many host proteins induced by IFN-α/β inhibit PRRSV replication, such as TRIM21 [23], OAS1b [24], PKR [25], and so on. To survive in the host, PRRSV has evolved multiple strategies to antagonize host innate immune responses. Previous studies have reported that PRRSV N interferes with RIG-I ubiquitination by interacting with TRIM25 to antagonize the type I interferon signaling pathway [20]. Herein, our study first reports that swine NONO promotes IFN-β expression by interacting with IRF3 to restrict PRRSV infection. Furthermore, NONO associates with PRRSV N protein and IRF3 to enhance phosphorylation of IRF3. Mechanistically, NONO recruits N protein and IRF3 to form N-NONO-IRF3 complex to inhibit PRRSV propagation. Interestingly, it was found that NONO protein reverses the inhibitory effect of PRRSV N protein on the type I IFN signaling pathway.

As an RNA virus, the PRRSV genome is recognized by retinoic acid-inducible gene I (RIG-I)-like receptors (RLRs) in the cytosol. RLRs contain three members: RIG-I, melanoma differentiation-associated protein 5 (MDA5), and laboratory of genetics and physiology 2 (LGP2). Activation of RIG-I and MDA5 recruits mitochondrial anti-viral signaling protein (MAVS; also named VISA, IPS-1) to promote phosphorylation of TANK-binding protein-1 (TBK1). After interaction with TBK1, interferon regulatory factor (IRF3) homodimerizes and translocates to the nucleus and increases IFN-β expression to inhibit viral replication [21,26,27]. NONO is a multifunctional nuclear protein involved in many important biological processes [10]. However, the function of NONO on the innate immune response has remained under characterized, except for its function as a sensor to promote cGAS-mediated innate immune activation by binding to HIV capsid [13]. In the present study, we provided evidence that NONO promoted IFN-β production and inhibited PRRSV replication (Figs 1 and 2). Next, we identified IRF3 was the key adaptor molecule influenced by NONO in IFN-β signaling pathway. The results of AP-MS and co-IP experiments suggested that there was interaction between NONO and IRF3 (Figs 3 and S3A). In addition, NONO positively regulated poly (I:C)- and IRF3-induced IFN-β expression and IFN-β promoter activity (Figs 7 and 8). By Western blotting analysis, it was shown that NONO promoted phosphorylation of IRF3 induced by poly (I:C) and TBK1 (Fig 9). These data suggest that NONO, as a positive regulatory factor, enhances IRF3 activity to increase IFN-β expression. Meanwhile, PRRSV N protein inhibits RIG-I- and MAVS-mediated IFN-β expression and instead promotes TBK1- and IRF3-mediated IFN-β expression. However, N(NLS) mutant which only located in cytoplasm failed to promote IFN-β expression induced by TBK1 and IRF3, suggesting that N protein in nucleus binds to NONO and probably increases stability of phosphorylated IRF3 to promote type I IFN signaling pathway activity. In addition, NONO is a multifunctional nuclear protein to regulate many important biological processes, such as DNA repair, mRNA splicing, nuclear retention of defective RNA, and transcriptional regulation [10–12]. PRRSV N binds to NONO and IRF3 to form N-NONO-IRF3 complex and probably affects mRNA splicing levels of IFN-β and ISGs to regulate the activation of type I IFN signaling pathway, which needs to be further studied in the future.

**Fig 10:**
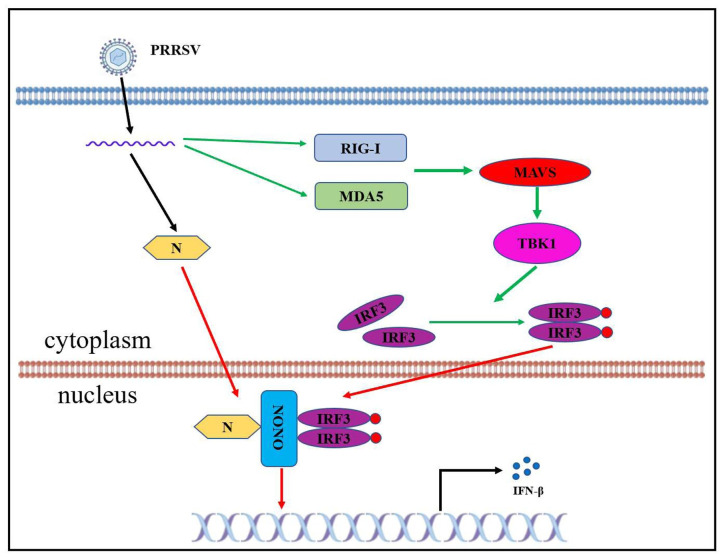
The working model for NONO positively regulating IRF3-mediated IFN-β signaling pathway by detection of PRRSV N.

PRRSV N protein is a small protein containing 128 and 123 amino acids in genotypes I and II, respectively. N protein is homodimerized and binds to RNA and plays an essential function in the virus life cycle [28,29]. In PRRSV-infected cells, N protein is not only distributed in the cytoplasm but also in the nucleus and nucleolus [30,31]. The nuclear localization of N protein is dependent on its NLS motif through binding to importin-α and importin-β [32,33]. The viral titers of NLS-null PRRSV which generates mutants in NLS of N protein are significantly lower than those of WT PRRSV, but produces the higher titers of neutralizing antibodies and shorter mean duration of viremia [34,35]. In addition, PRRSV N protein binds to TRIM25 to antagonize RIG-I-mediated IFN-β expression [20,36]. However, the function of PRRSV N protein in the nucleus on IFN-β signaling pathway is still unclear. We have identified that viral proteins regulated by NONO after PRRSV infection and found that NONO specially interacted and colocalized with PRRSV N protein in the nucleus, which further promoted the mRNA level of IFN-β, IFN-β promoter and PRD I-III promoter activities. As N(NLS) was obtained by K43G and K44G specific mutations which led to N protein localizing in the cytoplasm and there was no interaction between N(NLS) and NONO, K43 and K44 were the key sites for interaction between NONO and PRRSV N protein. (Figs 4–6). Interestingly, we found that PRRSV N protein positively regulated the mRNA level of IFN-β, IFN-β promoter and PRD I-III promoter activity induced by poly (I:C) or IRF3 in NONO expressing cells (Figs 7, 8 and S7), suggesting NONO protein reverses the inhibitory effect of PRRSV N protein on type I IFN signaling pathway reported by Zhao et al [20]. Our studies demonstrated that NONO increased phosphorylation of IRF3 and interaction with IRF3 through binding to PRRSV N protein (Fig 9), leading a work model that NONO, N and IRF3 to form N-NONO-IRF3 complex which then enhance the activation of IFN-β signaling pathway in the nucleus (Fig 10).

In summary, the results of our experiments describe for the first time a novel mechanism for PRRSV N protein mediated-activation of IFN-β signaling pathway in the nucleus. It is shown that NONO positively regulates IFN-β signaling pathway by targeting IRF3. Mechanistically, NONO functions as a scaffold protein to recruit PRRSV N protein and form N-NONO-IRF3 complex in the nucleus to exert an antiviral response. This study provides a new insight into antiviral immune response mediated by the host protein NONO which detects viral nucleocapsid protein and functions as a positive regulatory factor of IRF3.

## Materials and methods

### Cells, viruses, and chemicals

HEK-293T cells and MARC-145 cells were cultured in Dulbecco’s modified Eagle’s medium (DMEM) containing 10% fetal bovine serum (FBS; Gibco, Grand Island, USA), penicillin (100 U/mL), and streptomycin (100 μg/mL). PAM-KNU cells are immortalized porcine alveolar macrophages (PAMs) and maintained in RPMI 1640 medium supplemented with 5% FBS as previously described [37]. The sNONO KO-PAM-KNU cells with NONO knockout in PAM-KNU cells were generated by the CRISPR-Cas9 gene editing system and used previously to study the functions of NONO in our laboratory [38]. All cells were cultured in a humidified incubator with 5% CO_2_ at 37°C. HP-PRRSV SY0608 strain (GenBank: EU144079) was propagated, titrated in MARC-145 cells, and stored at -80°C until used [15]. Poly (I:C) (Sigma-Aldrich, St. Louis, MO, USA), which is a surrogate for viral dsRNA, was used for transfection of cells to activate the IFN-β signaling pathway.

### Plasmid, siRNA and transfection

The swine NONO gene (GenBank accession no. GACC01000054.1) was amplified from the cDNA of PAM-KNU cells using reverse transcription-PCR (RT-PCR) by SuperScript III reverse transcriptase (Invitrogen, Carlsbad, CA, USA) and inserted into pXJ41 to obtain plasmid pXJ41-sNONO with a FLAG tag. The pXJ41-sNONO-NTD plasmid contains the N-terminal amino acids 58 to 217 of sNONO and pXJ41-sNONO-CTD plasmid contains the C-terminal amino acids 218 to 371 of sNONO [13,39]. The pXJ41-N-NTD and pXJ41-N-CTD plasmids contain the N-terminal amino acids 1 to 56 of N and C-terminal amino acids 57 to 123 of N, respectively. Specific mutations (K43G and K44G) were introduced to pXJ41-N and pXJ41-N(NLS) plasmid was obtained with slight modifications as described elsewhere [40–42]. The PRRSV N related expression plasmids were stored in our lab. The plasmids pXJ41-hRIG-I, pXJ41-hMAVS, pXJ41-hTBK1, or pXJ41-hIRF3 contained human genes with a MYC tag were kindly provided by Chengjiang Gao, Shandong University, Jinan, China. The plasmids pXJ41-N contained N gene of HP-PRRSV SY0608 with an HA tag and pXJ41-sRIG-I, pXJ41-sMAVS, pXJ41-sTBK1, or pXJ41-sIRF3 contained swine genes with a MYC tag were generated and stored in our laboratory. The primers used in this study are listed in Table 1. The siRNA sequence to knockdown sNONO expression were as follows: 5’-CCAGAAUGAAGGCUUAACUTT-3’, 5’-AGUUAAGCCUUCAUUCUGGTT-3’. For SLCA, the LN-nsp1α, LN-nsp1β, LN-nsp2 to LN-nsp12, LN-ORF2a, LN-ORF2b, LN-GP3, LN-GP4, LN-GP5, LN-GP5a, LN-M, or LN-N was generated and stored in our laboratory [43]. Lipofectamine 2000 reagents (Invitrogen) were used to transfect plasmids and poly (I:C) into cells. The siRNA was transfected into PAM-KNU cells using Lipofectamine RNAiMAX reagents (Invitrogen) according to the manufacturer’s instructions.

**Table 1:** Primers used in this study.

Primer name	Sequence (5’-3’) ^a^	Restriction site (promoter or tag)	Purpose
sNONO-Fwd	CGCGAATTCCCACCATG**GATTACAAGGATGACGACGATAAG**ATGCAGAGCAATAAAACT	*Eco*R I (FLAG)	sNONO amplification
sNONO-Rev	CGCAAGCTTTCATTAGTATCGGCGACGTTTGTTTGGA	*Hin*d III	
sNONO-NTD-Fwd	CGCGAATTCCCACCATG**GATTACAAGGATGACGACGATAAG**AAGAATTTTAGGAAACCAGGAGAGA	*Eco*R I (FLAG)	sNONO-NTD amplification
sNONO-NTD-Rev	CGCAAGCTTTCAAGGAAATGTGGTTAGCAGGAAGGAG	*Hin*d III	
sNONO-CTD-Fwd	TATGAATTCCCACCATG**GATTACAAGGATGACGACGATAAG**AGGCCTGTGACTGTGGAG	*Eco*R I (FLAG)	sNONO-CTD amplification
sNONO-CTD-Rev	CGCAAGCTTTAGGTTCCCTTGAATCCTTCCTG	*Hin*d III	
LC-sNONO-Fwd	CATCATCTCGAGGCCACCATGCAGAGCAATAAAACTT	*Xho* I	sNONO amplification
LC-sNONO-Rev	AGAAGAGCGGCCGCTCATTAGTATCGGCGACGTTTGTTT	*Not* I	
β-actin-Fwd	TCTGGCACCACACCTTCT		β-actin amplification
β-actin-Rev	GATCTGGGTCATCTTCTCAC		
sNONO-RT-Fwd	AGCGGAGATTGCCAAAGTG		sNONO amplification
sNONO-RT-Rev	GGAAGGTTTCGGACCGTAAG		
PRRSV-N-RT-Fwd	TGTGCCAAATGCTGGGTA		PRRSV N amplification
PRRSV-N-RT-Rev	GGGTAAAGTGATGCCTGACG		
sISG15-RT-Fwd	GCAATGTGCTTCAGGATGG		sISG15 amplification
sISG15-RT-Rev	AGGCTTGAGGTCATACTCCC		
sIFN-β-RT-Fwd	TGCATCCTCCAAATCGCTCT		sIFN-β amplification
sIFN-β-RT Rev	ATTGAGGAGTCCCAGGCAAC		
sMx1-RT-Fwd	GAAGGGCAAAGTCAGTTACC		sMx1 amplification
sMx1-RT-Rev	CAGGAAGGTCTATGAGGGTC		

^a^Restriction endonuclease sites are underlined. The FLAG tag is in boldface.

### Virus titers

Cells were seeded in 12-well plates (5 × 10^5^ cells per well). 24 h later, cells were transfected with indicated plasmids and infected with PRRSV at an MOI of 1. The culture supernatants were harvested at indicated times. Half of supernatants was subjected to microtitration infectivity assay on MARC-145 cells, and the titers were calculated for 50% tissue culture infective dose (TCID_50_) using the Reed-Muench method. The other half of the supernatants was analyzed by real-time PCR.

### Real-time PCR

Cells were seeded in 12-well plates (5 × 10^5^ cells per well). 24 h later, cells were transfected with indicated plasmids and infected with PRRSV at an MOI of 1 or treated with 1 μg/mL poly (I:C). Total RNA was extracted by TRIzol reagent (Invitrogen) according to the manufacturer’s instructions. The cDNA was synthesized by PrimeScript RT reagent kit with gDNA Eraser (TaKaRa, Tokyo, Japan) and used for SYBR green PCR assay (TaKaRa). The abundance of individual mRNA transcripts in each sample was assayed three times and normalized to the level of human glyceraldehyde-3-phosphate dehydrogenase (GAPDH) mRNA (as an internal control). Relative transcript levels were quantified by the 2^-ΔΔCT^ (where CT is threshold cycle) method and are shown as relative expression change.

The viral RNA in the culture supernatants was extracted by TRIzol reagent and reverse transcribed to cDNA by PrimeScript RT reagent kit with gDNA Eraser according to the manufacturer’s instructions. The pXJ41-N plasmid was tenfold serially diluted and used to generate a standard curve. PRRSV cDNA copies of samples were determined by linear extrapolation of the CT value plotted against the standard curve. All assays were repeated at least three times, with each experiment performed in triplicate. Primers used in this study are listed in Table 1.

### Co-immunoprecipitation (Co-IP) assay

Cells were seeded in 60 mm cell culture dishes (2.6 × 10^6^ cells per dish) for 24 h and transfected with indicated plasmids. Cells were collected and lysed in lysis buffer containing 1.0% (vol/vol) NP-40, 50 mM Tris-HCl, pH 7.4, 50 mM EDTA, 150 mM NaCl, and a protease inhibitor PMSF. After centrifugation for 10 min at 12,000 g, supernatants were collected and incubated with specific antibodies overnight, and then protein A/G beads (Santa Cruz Biotechnology) were added. After 8 h of incubation, beads were washed five times with lysis buffer containing PMSF. The samples were analyzed by SDS-PAGE and Western blotting.

### Western blotting

HEK-293T and PAM-KNU cells were seeded in 6-well plates (1 × 10^6^ cells per well). 24 h later, cells were transfected with indicated plasmids and treated with 1 μg/mL poly (I:C). Cells were lysed in lysis buffer supplemented with phenylmethylsulfonyl fluoride (PMSF; Beyotime, China). The samples were analyzed by sodium dodecyl sulfate-polyacrylamide gel electrophoresis (SDS-PAGE) and Western blotting. Separated proteins were then transferred onto a nitrocellulose membrane and probed with antibody (Ab) against FLAG (Sigma-Aldrich), HA (Sigma-Aldrich), NONO (Santa Cruz Biotechnology, Santa Cruz, CA, USA), ISG15 (GeneTex), MYC (Sigma-Aldrich), IRF3 (Cell Signaling Technology), p-IRF3 (Ser396, Cell Signaling Technology), PRRSV N (kept in our lab) and β-actin antibody (Boster, Wuhan, China). Specific reaction products were detected with horseradish peroxidase (HRP)-conjugated goat anti-rabbit IgG and goat anti-mouse IgG (Boster, Wuhan, China). The membranes were developed using SuperSignal WestPico Chemiluminescent Substrate according to the manufacturer’s suggestions (Pierce, Rockford, IL, USA).

### Indirect immunofluorescence

PAM-KNU cells were seeded directly onto coverslips in 24-well plates (2.5 × 10^5^ cells per well) for 24 h and transfected with plasmids pXJ41-sNONO, pXJ41-N, pXJ41-N(NLS), and pXJ41 for 18 h or infected with PRRSV at an MOI of 1 for 12 h. Cells were washed three times in ice-cold phosphate-buffered saline (PBS) and fixed with 4% paraformaldehyde in PBS for 30 min. After three times washed, cells were permeabilized with 0.5% Triton X-100 for 15 min. The coverslips were incubated with antibodies against FLAG, HA, and PRRSV N diluted in PBS for 1 h. After washing three times with PBS, coverslips were incubated with Alexa Fluor 488-conjugated goat anti-mouse IgG(H+L) and Alexa Fluor 594-conjugated goat anti-rabbit IgG(H+L) antibodies at room temperature for 1 h. Then, coverslips were incubated with 4’,6’-diamidino-2-phenylindole (DAPI; Sigma-Aldrich), mounted with antifade mounting medium (Beyotime, China), and observed under an Olympus BX51 inverted fluorescence microscope.

### Luciferase reporter gene assay

PAM-KNU and HEK-293T cells were seeded in 12-well plates (5 × 10^5^ cells per well) for 24 h and transfected with indicated plasmids containing the IFN-β promoter (pIFN-Luc) or PRD I-III promoter (pPRDI-III-Luc) along with pRL-TK luciferase reporter plasmids. Luciferase activities were measured using a dual-luciferase reporter assay kit (Promega, Madison, WI, USA) according to the manufacturer’s protocol. The values were normalized with respect to *Renilla* luciferase activities. Then the results were expressed as relative luciferase activities, which are shown as fold change relative to the mock-treated control untransfected cells. All assays were repeated at least three times, with each experiment performed in triplicate.

### Split luciferase complementation assay (SLCA)

HEK-293T cells were seeded in 12-well plates (5 × 10^5^ cells per well) for 24 h and cotransfected with LC-sNONO and LN-PRRSV plasmids. The ZIKV C protein expression plasmids (LN-C and LC-C) which were confirmed to be interactive between them were used as a positive control as previously described [44], whereas the pXJ41 empty vector was used as a negative control. At 24 h posttransfection, cells were lysed by lysis buffer and clarified by centrifugation at 12,000 × g for 10 min at 4°C. Relative luciferase units (RLU) were detected using luciferase assay system (Promega) according to the manufacturer’s protocol [45].

### Statistical analysis

Data were compared and the differences were determined by one-way repeated measurement analysis of variance [46] and least significance difference (LSD). A *P* value of < 0.05 was considered statistically significant [47].

## Supporting information

S1 Fig Related to Fig 1. NONO inhibits PRRSV replication in MARC-145 cells. (A-D) MARC-145 cells were transfected with 1 μg of pXJ41-sNONO or pXJ41 for 24 h and infected with HP-PRRSV SY0608 strain at an MOI of 1. Culture supernatants were collected at indicated times. Virus titers in culture supernatants were measured by microtitration infectivity assay and calculated TCID_50_ using the Reed-Muench method (A) (n = 3 independent experiment, **p < 0.01, bar indicates mean). Viral RNA was extracted from culture supernatants and analyzed by real-time PCR (B) (n = 3 independent experiment, *p < 0.05, **p < 0.01, bar indicates mean). PRRSV N protein expression was detected by mouse anti-N antibody after PRRSV infection. The same blot was incubated with β-actin antibody as a protein loading control (C) (n = 3 independent experiment, one representative experiment is shown). The band intensities of N are shown as the relative protein expression levels, normalized with β-actin (D) (n = 3 independent experiment, *p < 0.05, **p < 0.01, bar indicates mean).
(TIF)

S2 Fig Related to Fig 2. NONO up-regulates activation of IFN-β promoter induced by poly (I:C) or VSV. (A-C) PAM-KNU cells were transfected with 10 pmol of siNONO or siCtrl for 24 h, along with *Renilla* luciferase reporter and IFN-β promoter, and then treated with 1 μg/mL poly (I:C) or infected with VSV at an MOI of 0.1 for 6 h (A). PAM-KNU cells were transfected with 1 μg of pXJ41-sNONO or pXJ41 for 24 h, along with *Renilla* luciferase reporter and IFN-β promoter, and treated with 1 μg/mL poly (I:C) or infected with VSV at an MOI of 0.1 for 6 h (B). PAM-KNU and sNONO KO-PAM-KNU cells were transfected with *Renilla* luciferase reporter and IFN-β promoter and then treated with 1 μg/mL poly (I:C) or infected with VSV at an MOI of 0.1 for 6 h (C). Cells were harvested and IFN-β promoter activity was analyzed by a dual-luciferase reporter assay (n = 3 independent experiment, *p < 0.05, **p < 0.01, bar indicates mean).
(TIF)

S3 Fig Related to Fig 3. NONO interacts with IRF3 and fails to affect protein levels of IRF3 and p65. (A) HEK-293T cells were transfected with 6 μg of pXJ41-hIRF3 or pXJ41 for 24 h. Cells were harvested and subjected to co-IP with anti-MYC antibody. Immunocomplexes were analyzed by Western blotting using anti-MYC or anti-NONO antibody. WCL was also subjected to Western blotting using anti-MYC, anti-NONO, or anti-β-actin antibody (n = 3 independent experiment, one representative experiment is shown). (B) PAM-KNU and sNONO KO-PAM-KNU cells were seeded 6-well plates (1 × 10^6^ cells per well) for 24 h. Cells were lysed in lysis buffer and subjected to Western blotting using anti-IRF3, anti-p65, anti-NONO or anti-β-actin antibody (n = 3 independent experiment, one representative experiment is shown).
(TIF)

S4 Fig Related to Fig 4. NONO fails to affect the localization of PRRSV N protein. (A-B) PAM-KNU and sNONO KO-PAM-KNU cells were transfected with 0.5 μg of pXJ41-N (HA tag) for 18 h (A) or infected with PRRSV at an MOI of 1 for 12 h (B). Indirect immunofluorescence (IFA) was performed using anti-HA antibody (green), anti-N antibody (green) or DAPI (blue). Scale bar, 10 μm.
(TIF)

S5 Fig Related to Fig 8. The N(NLS) fails to regulate TBK1- and IRF3-mediated IFN-β expression. (A) PAM-KNU cells were transfected with 0.5 μg of pXJ41-N(NLS) or pXJ41 for 24 h, along with 0.5 μg of pXJ41-sRIG-I, pXJ41-sMAVS, pXJ41-sTBK1, or pXJ41-sIRF3. Cells were harvested and total RNA was extracted. The mRNA level of IFN-β was analyzed using real-time PCR (n = 3 independent experiment, *p < 0.05, **p < 0.01, "ns" stands for not statistically significant, bar indicates mean). Whole-cell lysates were immunoblotted with anti-MYC or anti-HA antibody. The same blot was incubated with β-actin antibody as a protein loading control (n = 3 independent experiment, one representative experiment is shown). (B) PAM-KNU cells were transfected with 0.3 μg of pXJ41-N(NLS) or pXJ41 together with 0.3 μg of pXJ41-sRIG-I, pXJ41-sMAVS, pXJ41-sTBK1, or pXJ41-sIRF3 with 0.3 μg of pIFN-β-Luc and 0.03 μg of pRL-TK luciferase reporter for 24 h. The activation of IFN-β promoter was detected using a dual-luciferase reporter assay (n = 3 independent experiment, **p < 0.01, "ns" stands for not statistically significant, bar indicates mean). Whole-cell lysates were immunoblotted with anti-MYC or anti-HA antibody. The same blot was incubated with β-actin antibody as a protein loading control (n = 3 independent experiment, one representative experiment is shown).
(TIF)

S6 Fig Related to Fig 8. PRRSV N protein does not directly bind to TBK1 or IRF3. HEK-293T cells were transfected with 3 μg of pXJ41-N and 3 μg of pXJ41-sTBK1 (A) or pXJ41-sIRF3 (B) for 24 h. Cells were harvested and subjected to co-IP with anti-HA antibody. Immunocomplexes were analyzed by Western blotting using anti-HA or anti-MYC antibodies. WCL was also subjected to Western blotting using anti-HA, anti-MYC, or anti-β-actin antibody (n = 3 independent experiment, one representative experiment is shown).
(TIF)

S7 Fig Related to Fig 8. NONO promotes activities of PRD I-III promoter through binding to PRRSV N protein. HEK-293T cells were cotransfected with 0.3 μg of pXJ41-sNONO and 0.3 μg of pXJ41-N, pXJ41-N(NLS) or pXJ41 together with 0.3 μg of pXJ41-sIRF3 for 24 h, along with 0.3 μg of pPRDI-III-Luc and 0.03 μg of pRL-TK luciferase reporter. Cells were harvested and PRD I-III promoter activity was analyzed by a dual-luciferase reporter assay (n = 3 independent experiment, **p < 0.01, "ns" stands for not statistically significant, bar indicates mean). Whole-cell lysates were immunoblotted with anti-MYC, anti-FLAG, or anti-HA antibody. The same blot was incubated with β-actin antibody as a protein loading control (n = 3 independent experiment, one representative experiment is shown).
(TIF)

S1 Table Candidate proteins analyzed by AP-MS, related to Fig 3B. (XLSX)

S1 Data The raw data supporting each of the manuscript Figures are contained in this Excel file. (XLSX)
