# Supplementary figures and images for: Swine NONO promotes IRF3-mediated antiviral immune response by Detecting PRRSV N protein
Source: PLoS Pathog. 2024 Oct 16;20(10):e1012622. doi: 10.1371/journal.ppat.1012622 (PMC11482726; doi:10.1371/journal.ppat.1012622)

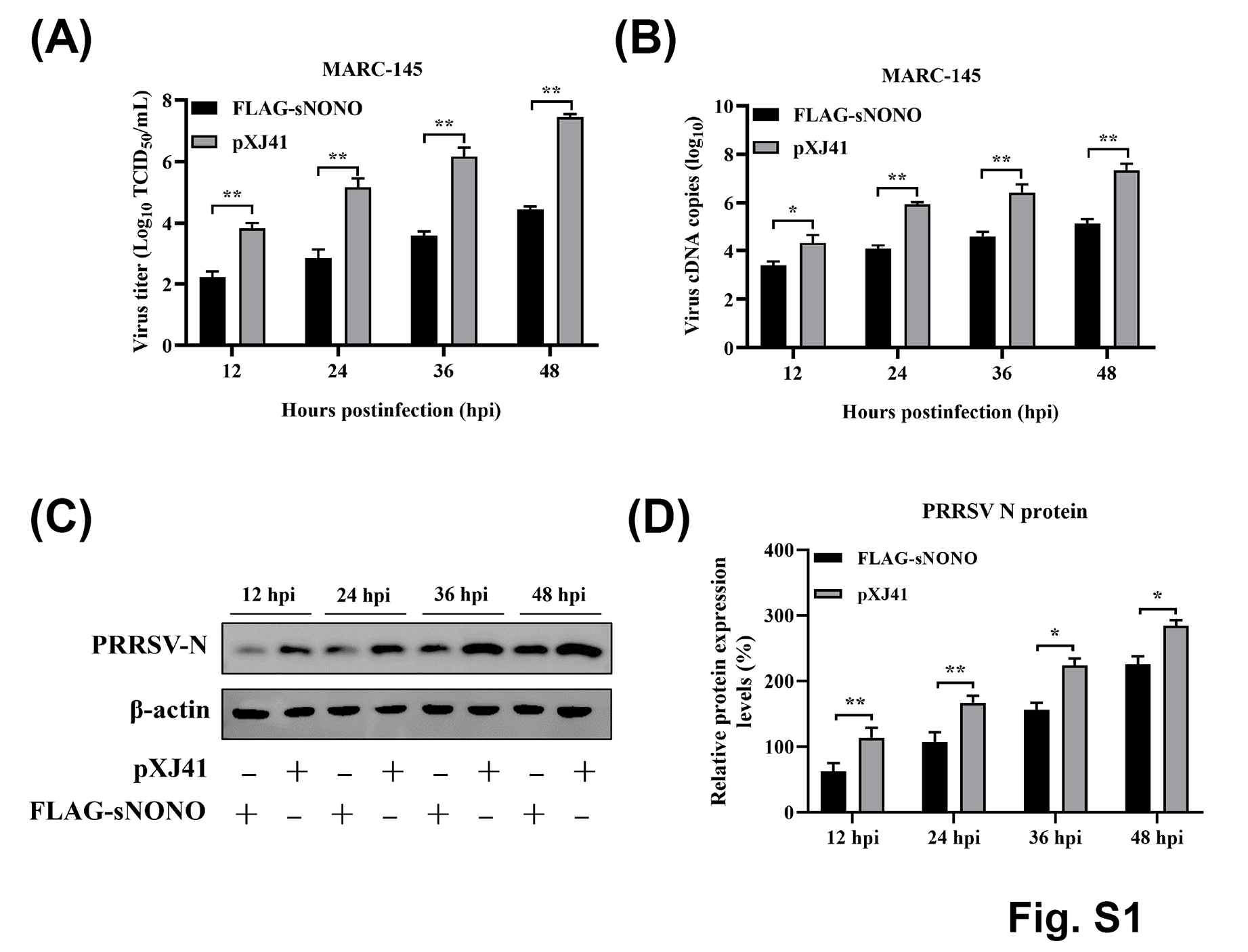

Supplement: S1 Fig — NONO inhibits PRRSV replication in MARC-145 cells. (A-D) MARC-145 cells were transfected with 1 μg of pXJ41-sNONO or pXJ41 for 24 h and infected with HP-PRRSV SY0608 strain at an MOI of 1. Culture supernatants were collected at indicated times. Virus titers in culture supernatants were measured by microtitration infectivity assay and calculated TCID50 using the Reed-Muench method (A) (n = 3 independent experiment, **p < 0.01, bar indicates mean). Viral RNA was extracted from culture supernatants and analyzed by real-time PCR (B) (n = 3 independent experiment, *p < 0.05, **p < 0.01, bar indicates mean). PRRSV N protein expression was detected by mouse anti-N antibody after PRRSV infection. The same blot was incubated with β-actin antibody as a protein loading control (C) (n = 3 independent experiment, one representative experiment is shown). The band intensities of N are shown as the relative protein expression levels, normalized with β-actin (D) (n = 3 independent experiment, *p < 0.05, **p < 0.01, bar indicates mean). (TIF) [file ppat.1012622.s001.tif]

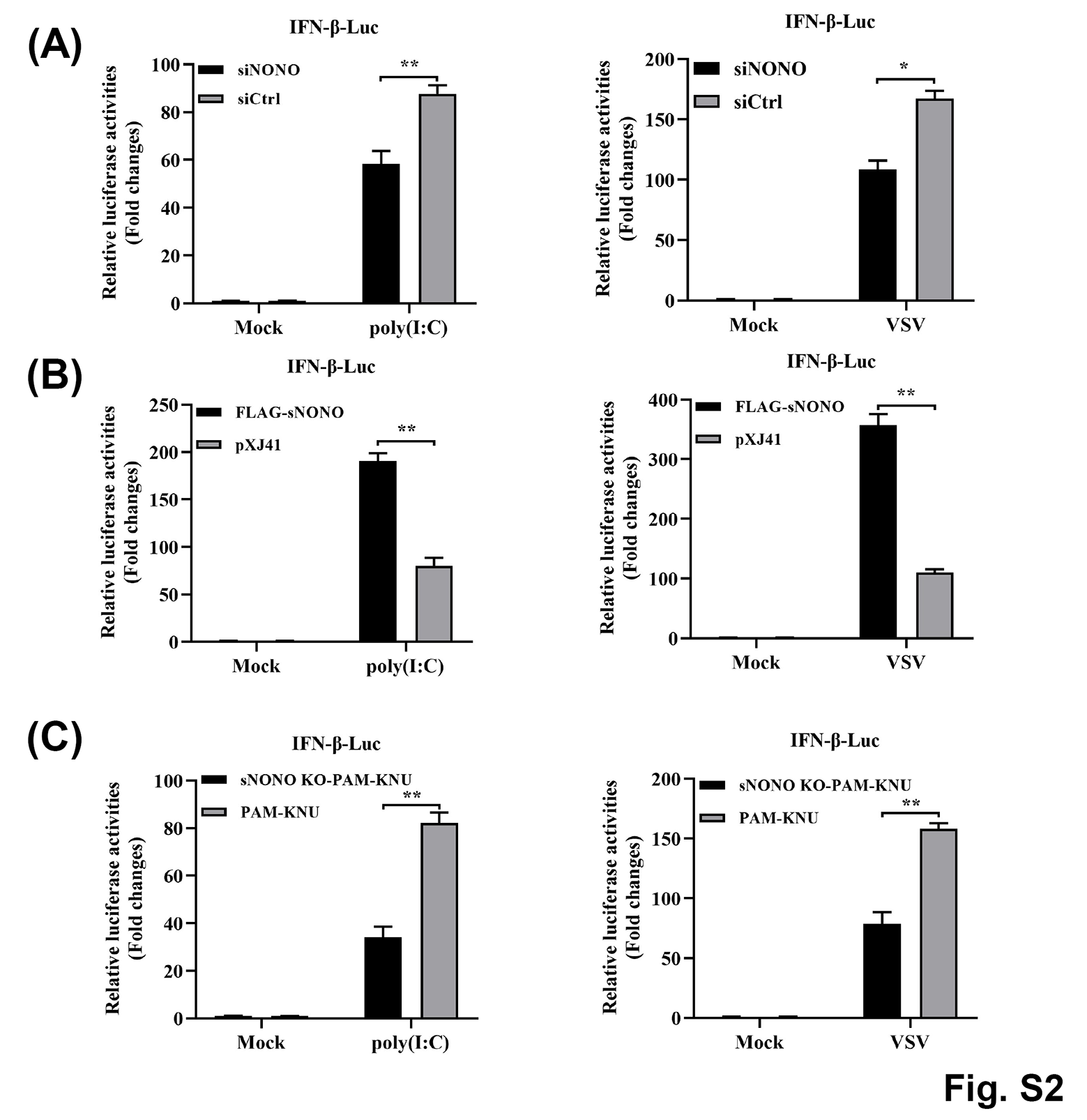

Supplement: S2 Fig — NONO up-regulates activation of IFN-β promoter induced by poly (I:C) or VSV. (A-C) PAM-KNU cells were transfected with 10 pmol of siNONO or siCtrl for 24 h, along with Renilla luciferase reporter and IFN-β promoter, and then treated with 1 μg/mL poly (I:C) or infected with VSV at an MOI of 0.1 for 6 h (A). PAM-KNU cells were transfected with 1 μg of pXJ41-sNONO or pXJ41 for 24 h, along with Renilla luciferase reporter and IFN-β promoter, and treated with 1 μg/mL poly (I:C) or infected with VSV at an MOI of 0.1 for 6 h (B). PAM-KNU and sNONO KO-PAM-KNU cells were transfected with Renilla luciferase reporter and IFN-β promoter and then treated with 1 μg/mL poly (I:C) or infected with VSV at an MOI of 0.1 for 6 h (C). Cells were harvested and IFN-β promoter activity was analyzed by a dual-luciferase reporter assay (n = 3 independent experiment, *p < 0.05, **p < 0.01, bar indicates mean). (TIF) [file ppat.1012622.s002.tif]

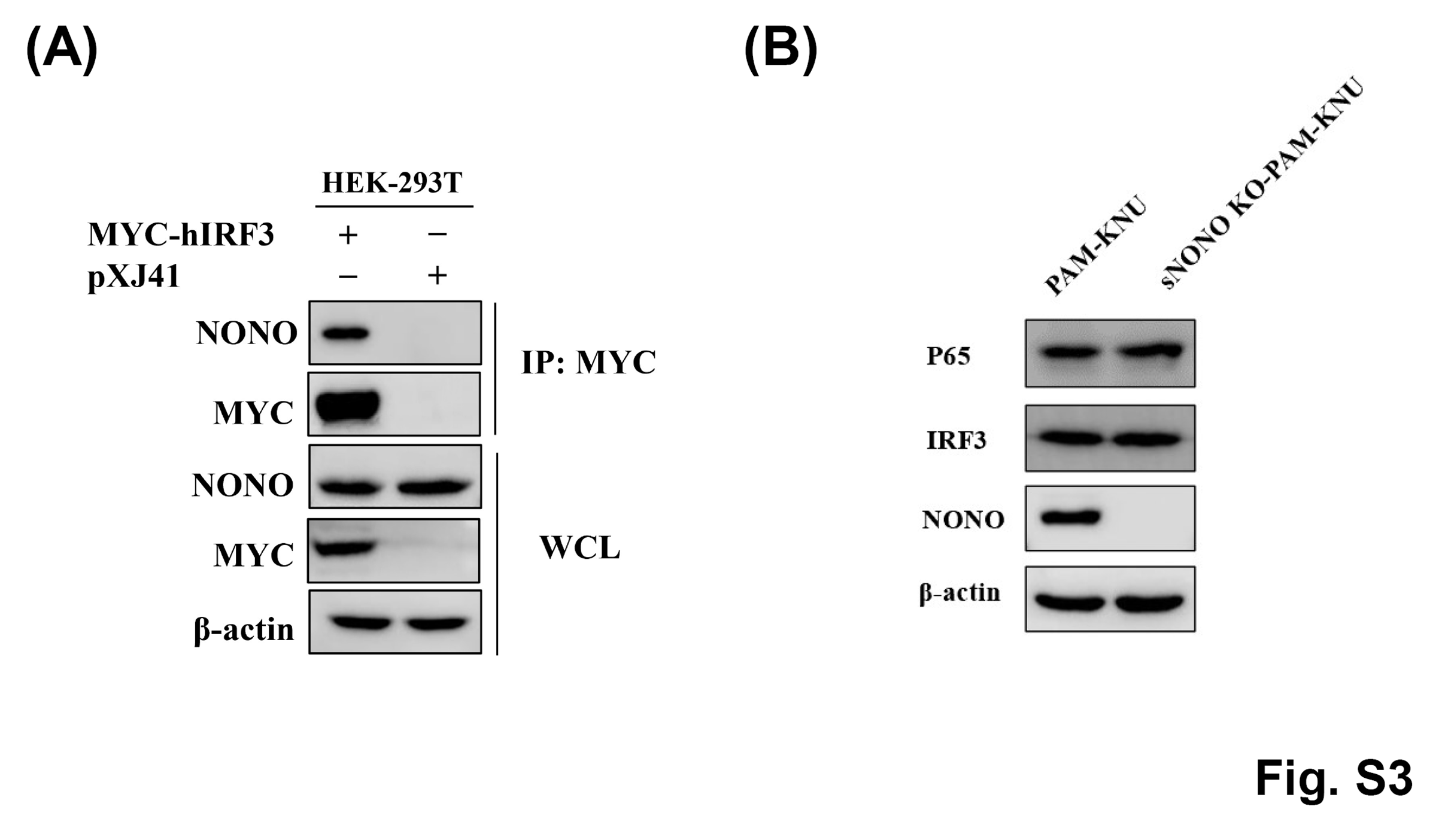

Supplement: S3 Fig — NONO interacts with IRF3 and fails to affect protein levels of IRF3 and p65. (A) HEK-293T cells were transfected with 6 μg of pXJ41-hIRF3 or pXJ41 for 24 h. Cells were harvested and subjected to co-IP with anti-MYC antibody. Immunocomplexes were analyzed by Western blotting using anti-MYC or anti-NONO antibody. WCL was also subjected to Western blotting using anti-MYC, anti-NONO, or anti-β-actin antibody (n = 3 independent experiment, one representative experiment is shown). (B) PAM-KNU and sNONO KO-PAM-KNU cells were seeded 6-well plates (1 × 10^6^ cells per well) for 24 h. Cells were lysed in lysis buffer and subjected to Western blotting using anti-IRF3, anti-p65, anti-NONO or anti-β-actin antibody (n = 3 independent experiment, one representative experiment is shown). (TIF) [file ppat.1012622.s003.tif]

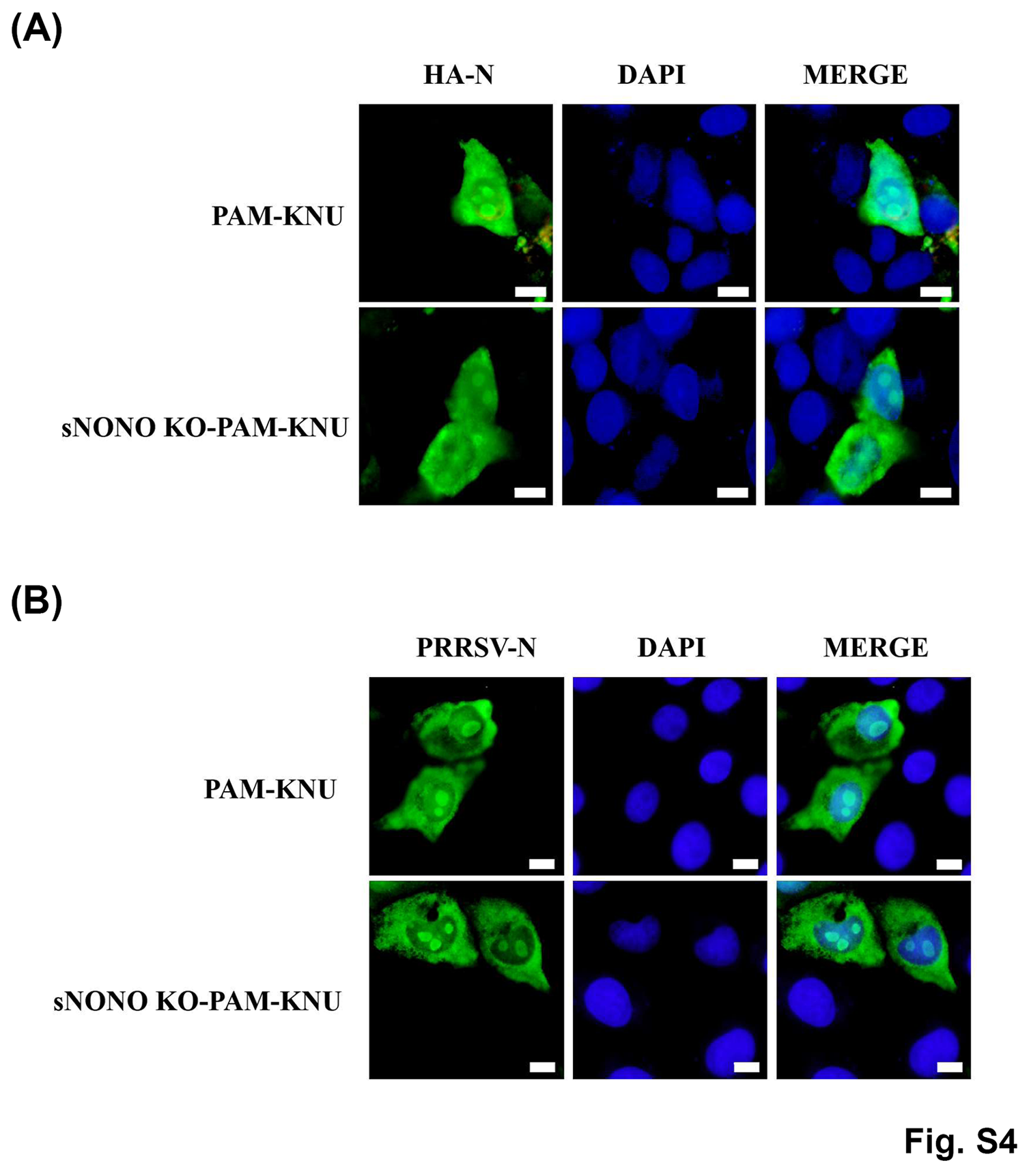

Supplement: S4 Fig — NONO fails to affect the localization of PRRSV N protein. (A-B) PAM-KNU and sNONO KO-PAM-KNU cells were transfected with 0.5 μg of pXJ41-N (HA tag) for 18 h (A) or infected with PRRSV at an MOI of 1 for 12 h (B). Indirect immunofluorescence (IFA) was performed using anti-HA antibody (green), anti-N antibody (green) or DAPI (blue). Scale bar, 10 μm. (TIF) [file ppat.1012622.s004.tif]

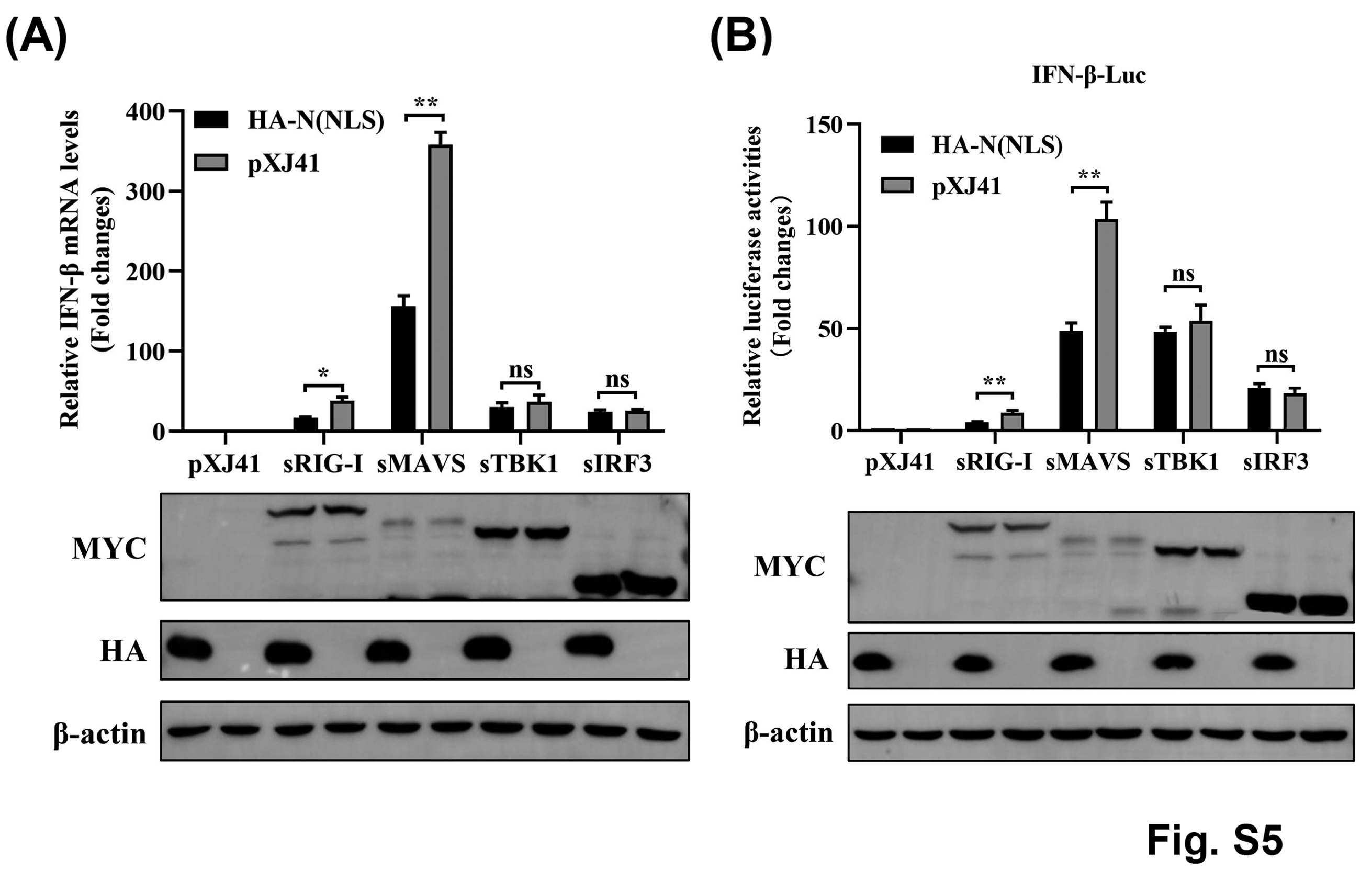

Supplement: S5 Fig — The N(NLS) fails to regulate TBK1- and IRF3-mediated IFN-β expression. (A) PAM-KNU cells were transfected with 0.5 μg of pXJ41-N(NLS) or pXJ41 for 24 h, along with 0.5 μg of pXJ41-sRIG-I, pXJ41-sMAVS, pXJ41-sTBK1, or pXJ41-sIRF3. Cells were harvested and total RNA was extracted. The mRNA level of IFN-β was analyzed using real-time PCR (n = 3 independent experiment, *p < 0.05, **p < 0.01, "ns" stands for not statistically significant, bar indicates mean). Whole-cell lysates were immunoblotted with anti-MYC or anti-HA antibody. The same blot was incubated with β-actin antibody as a protein loading control (n = 3 independent experiment, one representative experiment is shown). (B) PAM-KNU cells were transfected with 0.3 μg of pXJ41-N(NLS) or pXJ41 together with 0.3 μg of pXJ41-sRIG-I, pXJ41-sMAVS, pXJ41-sTBK1, or pXJ41-sIRF3 with 0.3 μg of pIFN-β-Luc and 0.03 μg of pRL-TK luciferase reporter for 24 h. The activation of IFN-β promoter was detected using a dual-luciferase reporter assay (n = 3 independent experiment, **p < 0.01, "ns" stands for not statistically significant, bar indicates mean). Whole-cell lysates were immunoblotted with anti-MYC or anti-HA antibody. The same blot was incubated with β-actin antibody as a protein loading control (n = 3 independent experiment, one representative experiment is shown). (TIF) [file ppat.1012622.s005.tif]

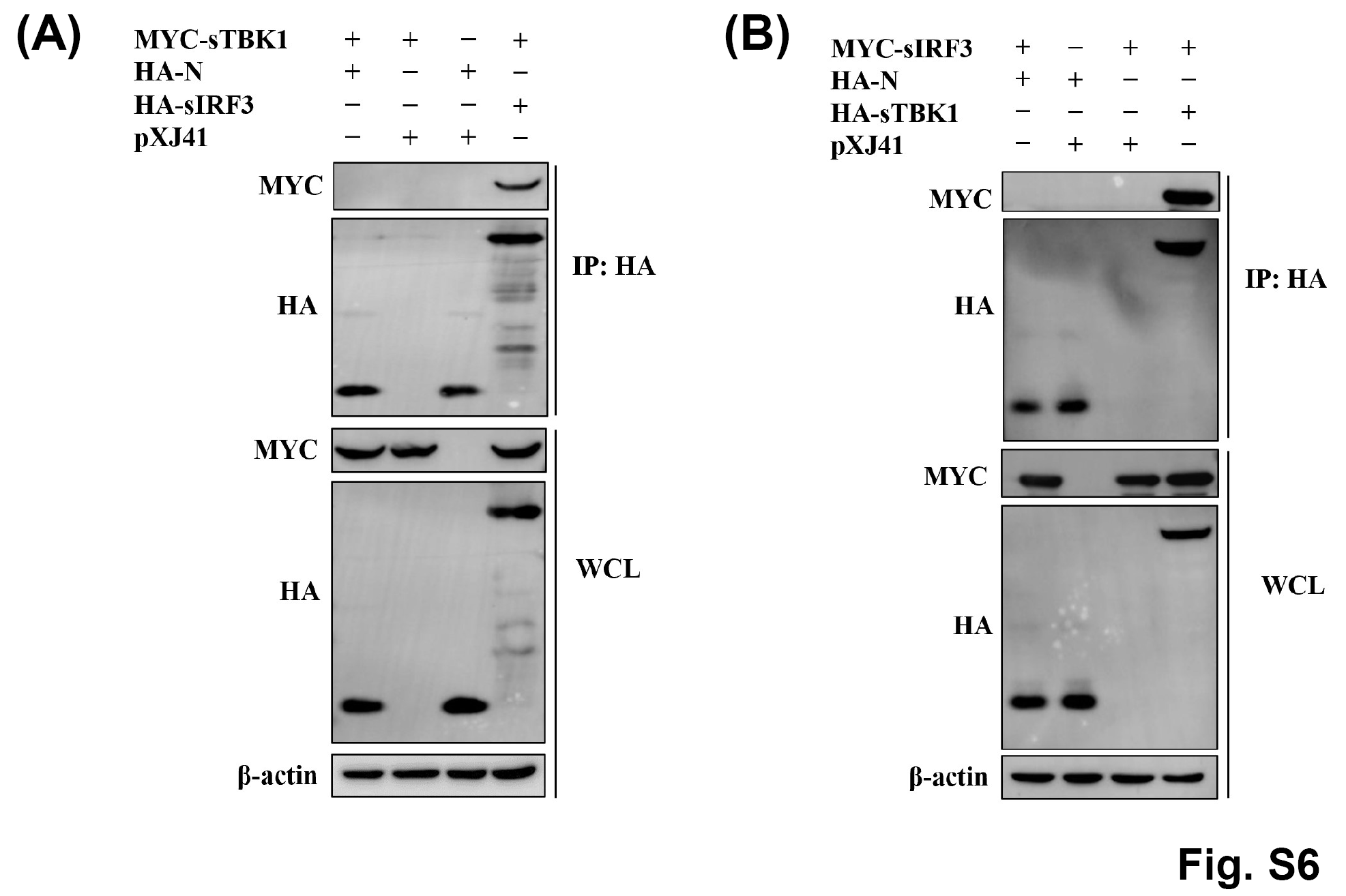

Supplement: S6 Fig — PRRSV N protein does not directly bind to TBK1 or IRF3. HEK-293T cells were transfected with 3 μg of pXJ41-N and 3 μg of pXJ41-sTBK1 (A) or pXJ41-sIRF3 (B) for 24 h. Cells were harvested and subjected to co-IP with anti-HA antibody. Immunocomplexes were analyzed by Western blotting using anti-HA or anti-MYC antibodies. WCL was also subjected to Western blotting using anti-HA, anti-MYC, or anti-β-actin antibody (n = 3 independent experiment, one representative experiment is shown). (TIF) [file ppat.1012622.s006.tif]

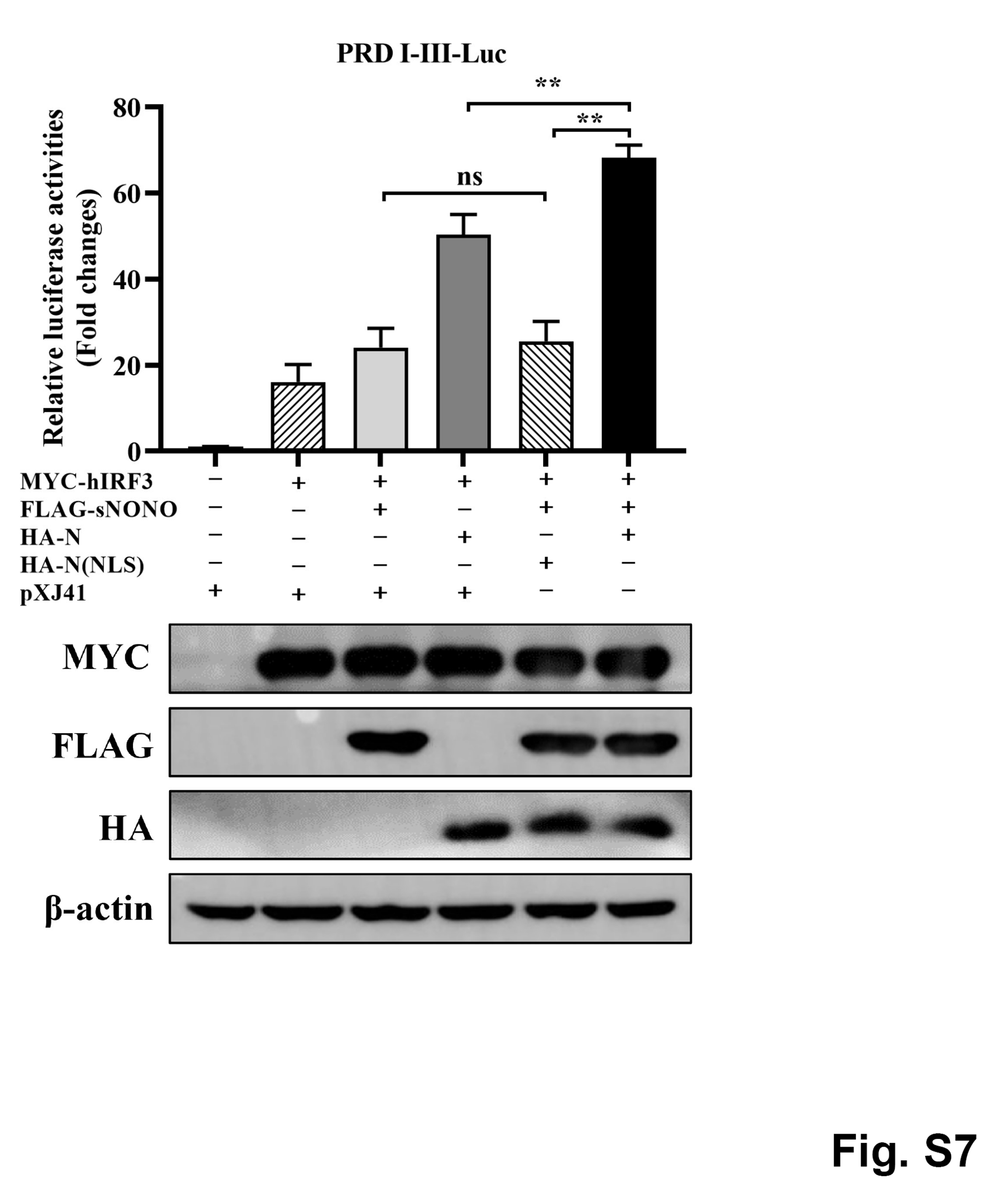

Supplement: S7 Fig — NONO promotes activities of PRD I-III promoter through binding to PRRSV N protein. HEK-293T cells were cotransfected with 0.3 μg of pXJ41-sNONO and 0.3 μg of pXJ41-N, pXJ41-N(NLS) or pXJ41 together with 0.3 μg of pXJ41-sIRF3 for 24 h, along with 0.3 μg of pPRDI-III-Luc and 0.03 μg of pRL-TK luciferase reporter. Cells were harvested and PRD I-III promoter activity was analyzed by a dual-luciferase reporter assay (n = 3 independent experiment, **p < 0.01, "ns" stands for not statistically significant, bar indicates mean). Whole-cell lysates were immunoblotted with anti-MYC, anti-FLAG, or anti-HA antibody. The same blot was incubated with β-actin antibody as a protein loading control (n = 3 independent experiment, one representative experiment is shown). (TIF) [file ppat.1012622.s007.tif]
